# LungElast—an open-source, flexible, low-cost, microprocessor-controlled mouse lung elastometer

**DOI:** 10.1038/s41598-023-38310-7

**Published:** 2023-07-12

**Authors:** Jesse D. Roberts

**Affiliations:** 1 Cardiovascular Research Center of the General Medical Services and the Departments of Anesthesia, Critical Care and Pain Medicine, Pediatrics, and Medicine, Massachusetts General Hospital - East, 149 13th St, Boston, MA USA; 2 Harvard Medical School, Harvard University, Cambridge, MA USA

**Keywords:** Respiration, Biomedical engineering

## Abstract

The study of mouse lung mechanics provides essential insights into the physiological mechanisms of pulmonary disease. Consequently, investigators assemble custom systems comprising infusion-withdrawal syringe pumps and analog pressure sensors to investigate the lung function of these animals. But these systems are expensive and require ongoing regulation, making them challenging to use. Here I introduce LungElast, an open-source, inexpensive, and self-contained instrument that can experimentally determine lung elasticity and volumes even in immature mice. It is assembled using custom 3D printed parts and readily available or easily constructed components. In this device, a microprocessor-controlled stepper motor automatically regulates lung volume by precisely driving a syringe piston whose position is determined using time-of-flight LIDAR technology. The airway pressures associated with the lung volumes are determined using compact sensor-on-chip technology, retrieved in a digital format, and stored by the microcontroller. The instrument software is modular, which eases device testing, calibration, and use. Data are also provided here that specify the accuracy and precision of the elastometer’s sensors and volume delivery and demonstrate its use with lung models and mouse pups. This instrument has excellent potential for research and educational work.

## Introduction

Elasticity is a fundamental physiologic property of lung tissue that affects its function^1–3^. During inflation with gas, the lung resists distention and stores kinetic energy in an extracellular network of elastin and collagen fibers, proteoglycans, and other molecules, and airway pressure increases. During lung deflation, the elastic potential energy is retrieved, which helps expel gas from the airways. The elasticity of the lung changes throughout pulmonary development due to extracellular matrix protein maturation and the alveolar formation^4,5^. Studies also show that aging and pathophysiologic mechanisms of disease dysregulate lung elasticity^6–14^. Accordingly, there is increasing development of tools that test how lung elasticity might be regulated in health and disease.

At a macro mechanical level, lung elasticity is determined experimentally by testing how changes in lung gas volumes affect airway pressures^15,16^. By convention, *full* volume-pressure (V-P) data are obtained by measuring the airway pressures that result from introducing metered volumes of gas into a deflated lung until it reaches the limit of its distensibility, often referred to as total lung capacity (TLC). Then, airway pressures continue to be determined while gas is withdrawn from the lung until it collapses, defined as residual volume (RV). *Partial* V-P data are acquired while determining these V-P relationships in a lung that is not inflated to TLC or deflated to RV. In both cases, the lung volumes can be changed using the intermittent or continuous movement of gas. Through mathematical modeling of the V-P data and determining the lung volumes associated with critical airway pressures, lung elastance and other parameters of physiologic significance are determined. A list of these parameters, and their meaning, is provided in Supplementary Table S1, which can be accessed at an online repository^17^. This, and other supplementary information and materials, is shown in Table 1.

Increasingly, mice are used to study the mechanisms of pulmonary development and injury. This is because advanced knowledge of mouse biology and readily available tools that can manipulate mouse genotypes provide new opportunities to test processes that regulate the physiologic function of the lung. Also, the animal’s compact size eases its handling and diminishes the quantity of reagents required to test developmental and pathogenic processes. But the small size of mice causes significant technical challenges in the study of their pulmonary mechanics (reviewed in^18^). This includes difficulties in precisely metering small changes in lung gas volumes during the acquisition of V-P data. Because of this challenge, most studies of mouse lung mechanics to date have employed adult animals with acute-^13,19–21^ or newborn-onset of pulmonary disease^22–24^. However, a few lung mechanics studies have included immature mice^25–27^, and instruments are being developed to study lung elasticity and volumes even in newborn animals (e.g.^28^).

Mouse lung elasticity has been investigated using custom-made instruments assembled with commercially available syringe pumps and rodent ventilators. In these systems, incremental or continuous gas volumes are injected and removed from the airway using an airtight piston. The piston is either actuated manually^19,22^ or mechanically using infusion-withdrawal syringe pumps^13,20,28–31^. In ventilator systems, where high lung inflation rates might be desired, the movement of gas is controlled by motors that drive pistons through linkages or by solenoids that regulate the flow of diving gases^32–37^. Although the syringe pump systems have provided important information about mouse lung mechanics, they have several drawbacks. Their syringe pump systems can be challenging to use because they are not automated; during the experiments, they require precisely timed interventions by the user to regulate the gas volume change at target airway pressures^13,30^. Inconsistent control of the syringe pump by the user, when the lung reaches full distention or collapse, can cause spurious measurement of TLC and RV, respectively. Also, the measurement of lung mechanics in immature mice can be difficult. This is because most syringe pumps are not designed to meter the small amounts of gas required to make V-P measurements in such small animals.

The custom-made systems determine the airway pressures using manometers or analog pressure sensors. Although the pressure sensors can be accurate, their analog output is subject to electrical interference. Thus, they add the requirement of signal conditioners, analog-to-digital conversion (ADC) devices, and chart recorders, increasing the complexity and cost of the system. Also, these custom elastometers are not standardized. Variations in the performance of the components assembled by a particular lab might affect the robustness and reproducibility of the data. The sensors' precision, accuracy, and variability, and the devices’ volume delivery reproducibility are seldom reported. Commercially available rodent ventilator systems have been used to study lung mechanics of immature^25–27^ and adult mice^38,39^. Studies indicate that for key physiologic parameters, they produce mouse lung physiologic data like that obtained using syringe-pump devices^38^. But in some configurations, these ventilator systems can also measure viscoelastic properties and differences between positive and negative ventilation of the lung (e.g.^35^). Nevertheless, these ventilator systems are closed source and expensive, and not available for use by investigators with limited budgets.

Here I present an open-source and microprocessor-controlled lung elastometer (LungElast). The device is purpose-built, employing a compact stepper motor to drive a syringe piston to precisely regulate small changes in lung volumes and a digital sensor that measures airway pressures, thereby providing data that are used to determine key mechanical properties of mouse lungs. Moreover, easily manufactured parts and readily available components are used to construct LungElast. The components are relatively inexpensive; the total cost of the instrument is ~ $200 (itemized in the *Bill of Materials*). Its design, fabrication, testing, calibration, and use are detailed below and in online supplementary materials and demonstration videos^17^. I also describe the controlling software and data that validate the performance of its sensors and volume delivery. I demonstrate how the instrument measures the pulmonary mechanics of elastomeric models and mice.

## Results and discussion

### LungElast hardware design

In this work, LungElast refers to the embodiment of the hardware parts, sensors, discrete electronic components, and calibrating and controlling software that comprises the instrument. It was designed using open-source modeling software^40^. It was built using custom, additive-manufactured 3D-printed parts, a compact stepper motor, a gas-tight glass syringe, and readily available or easily constructed, inexpensive sensor, microprocessor, and digital control boards. To increase the flexibility and customization of the device, LungElast was designed to be calibrated and controlled using scripts that are converted to binary machine code by an open-source Python interpreter (MicroPython)(Table 1).

**Table 1:** LungElast supplementary information and materials.

File name	File type	License	Online Location
*Supplementary information*			
Tables S1–S4	pdf	Public domain	OSF
Figures S1–S4			
*Construction files*			
Bill of materials	pdf	Public domain	OSF
Schematic			
Sensor controller board wiring			
Pressure sensor breakout board wiring			
Pressure sensor breakout board construction			
Ribbon cable construction			
Elastomeric lung construction			
ESP32 MicroPython resources			
*Custom part manufacturing files*			
3D printable LungElast hardware and elastomeric lung connector models	stl	CC-BY-NC-SA 3.0	OSF
3D models for user modification	url	CC-BY-NC-SA 3.0	TinkerCad
*Software files*			
LungElast	mpy	MIT (see *license.txt* at OSF)	OSF
BusTester			
DistVolCal			
CompCal			
Customizer			
defaults			
*Software dependencies*			
boot	py	Public domain	OSF
main			
config			
secrets			
Vl6180_LE_200			
stepper			
pca9685			
stats_LE			
*Demonstration video files*			
LungElast operation	mp4	Public domain	OSF
DistVolCal operation			
Customizer operation			

**Figure 1:**
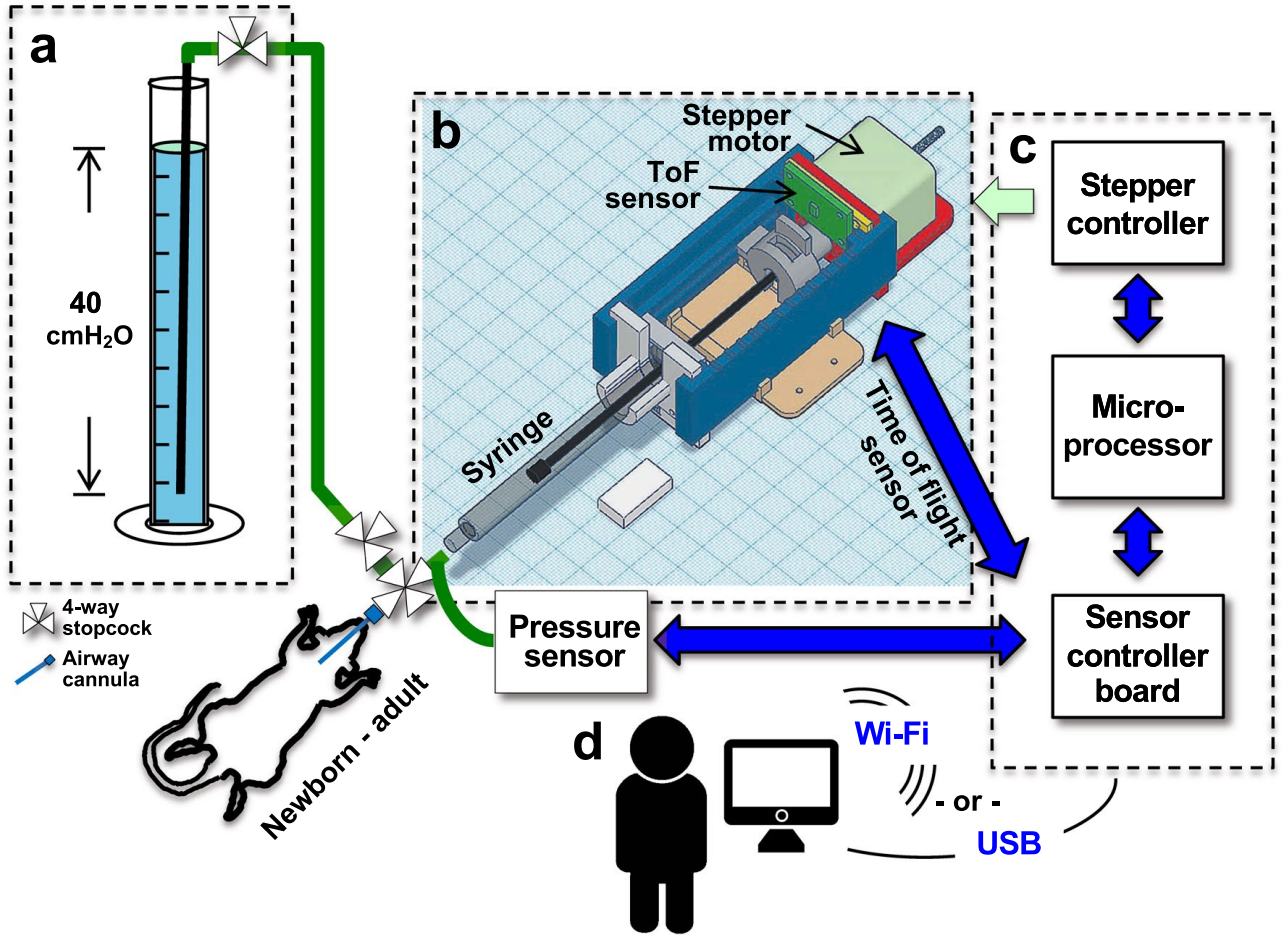
LungElast overview. (**a**) Pressure calibration apparatus. Water in a container with the end of the gas port 40 cm below its surface; 4-way stopcocks allow air to be injected or removed from the circuit during the pressure calibration. Blue: water; black and green: gas lines, rigid and flexible, respectively; white triangles: stopcocks. (**b**) 3D computer-aided design model of LungElast gas moving components, color-code is described in the following figure legend. The stepper motor, micro time-of-flight light detection and ranging proximity (ToF) sensor, and gas-tight syringe are also illustrated. Image attribution: TinkerCad. White scale: W10 × L20 × H5 cm. (**c**) Block diagram showing the relationship between the breakout boards harboring the stepper motor controller, ESP32 microprocessor, and the custom controller board for the ToF and pressure sensors. Blue lines: inter-integrated circuit (I^2^C) data bus; green line, the stepper motor coil wires. (**d**) User control. The user can load experiment parameters onto the LungElast microprocessor and initiate its automatic conduct of pulmonary elasticity and volume data experiments via hard-wired or Wi-Fi communications using a personal computer or smart device.

LungElast uses a microprocessor-controlled non-captive stepper motor to drive a lead screw that causes a syringe piston to inject or remove small aliquots of gas from a mouse lung while measuring the resulting airway pressures. A 3D rendering computer-aided design (CAD) model of the assembled LungElast hardware and an illustration of its external connections to a pressure calibration manometer and an animal subject is shown in Fig. 1. A block diagram is also provided showing the digital interconnections between the instrument’s electronic devices and the power connections to the stepper motor coils. It also illustrates the user interface with LungElast, via a USB hard-wired or Wi-Fi link.

During elastance experiments, the mouse is euthanized, and the lungs are allowed to collapse by elastic recoil in situ after a thoracotomy is performed^32^. In other cases, the animal is anesthetized, and the lungs collapse by absorption of alveolar O_2_ by the pulmonary circulation following an N_2_-washout^20,41,42^ before euthanasia. Then cannula is secured in the airway and it is connected to the instrument. In other cases, the lung can be dissected from the chest, collapsed by exposure to a vacuum if desired^19,42,43^, and connected to the elastometer^28,29^. However, in the latter case, care must be exercised not to puncture the lung during its extraction from the thorax. Figure S1 shows an experimental setup after a mouse pup was euthanized; the lungs were allowed to collapse, a cannula was secured in the airway, and it was connected to LungElast, as described in ‘Methods’ section. At this point in the experiment, the user executes the LungElast script using the device’s microprocessor, and they are instructed on how to calibrate the pressure sensor using the manometer. Then the device automatically obtains V-P data using the experiment’s parameters, which were previously set employing scripts described below.

**Figure 2:**
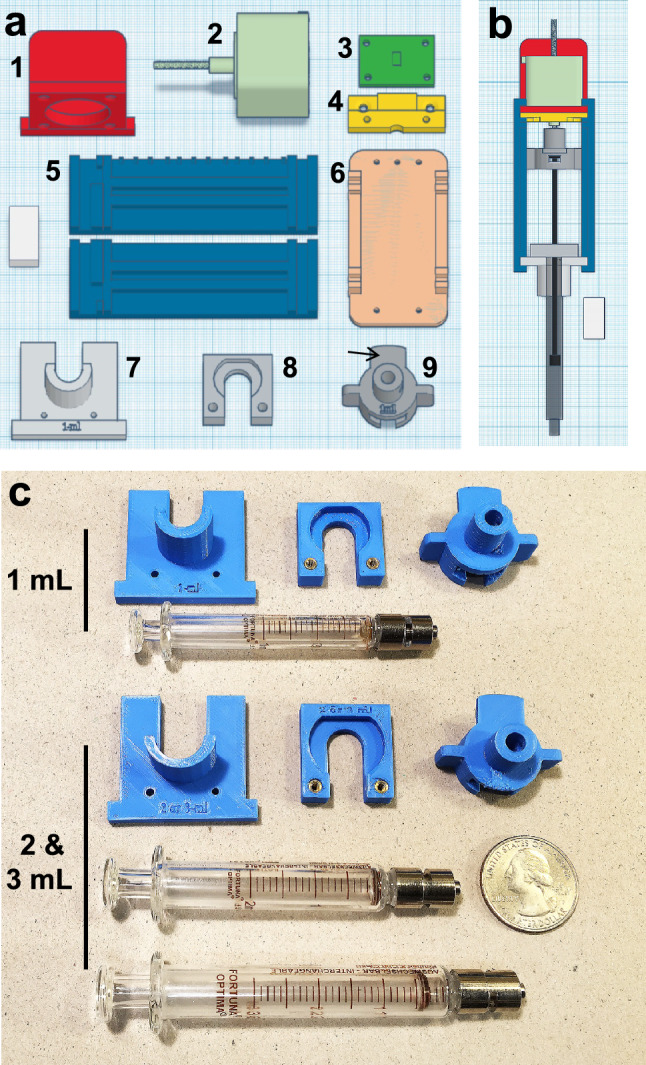
LungElast hardware components. (**a**) 3D CAD models of the LungElast mechanical elements: (1) stepper motor holder; (2) non-captive stepper motor; (3) ToF sensor breakout board; (4) ToF breakout board holder; (5) guides for lead screw nut, which is item 9 below, this guide has a 5 mm index incorporated on top; (6) guide base, optional; (7) syringe body rest; (8) syringe barrel flange lock; (9) lead screw nut with ToF sensor target (arrow). White scale: W10 × L20 × H 5 cm. Image attribution: TinkerCad. (**b**) Assembled mechanical elements show their integration and relationship. Image attribution: TinkerCad. (**c**) 3D prints of the parts that the user can customize to facilitate the assembly of a device that can accommodate 1–3 mL syringes for studying the lung mechanics of different-sized mice. Scale: 25-cent coin.

To provide a better understanding of the LungElast hardware, Fig. 2a,b show color-coded CAD models of the custom device parts and how they relate to each other in the assembly. The online supplementary materials provide standard tessellation language (STL) files for the 3D models of the LungElast hardware parts^17^. They include files for body, barrel, and piston flange holders that accommodate 1, 2, or 3 ml syringes. Manufactured examples of these later parts and the syringes are shown in Fig. 2c. Additionally, 3D CAD models of the lead screw nut guides are provided online at a repository^44^. After accessing them, the builder can modify the models and generate STL files that permit the manufacture of parts allowing the use of smaller or larger syringes with different gas movement capabilities if desired.

LungElast incorporates several design features in its gas-moving unit that enhance its utility. The device utilizes a small NEMA 11 bipolar stepper motor, increasing its compactness and decreasing its power requirement. The motor specified in the build drives a 2 mm pitch lead screw to finely control the syringe piston positioning and gas movement. It also has a holding torque of 8 N-Cm, which generates a force that is ~ sixfold excess of that required to drive a 1 ml syringe that was used to generate the pressures in the mouse pup lung V-P experiments. Moreover, the motor specified by the design employs a captive lead screw. Besides increasing the compactness of the instrument, this configuration allows the lead screw force vector to be directly aligned with the movement direction of the syringe piston. Compared with devices that employ eccentric control attachments^13^, this design feature should minimize the racking movement of the piston control assembly and thereby increase the piston's responsiveness.

**Figure 3:**
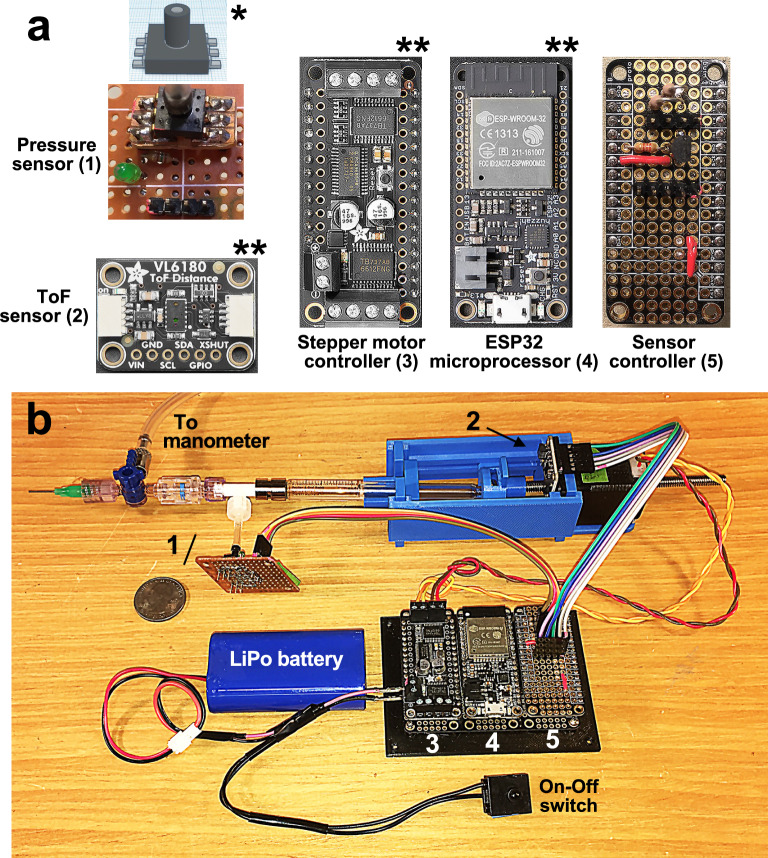
LungElast electronic components. (**a**) Sensor and controller breakout boards. LungElast is constructed using the indicated sensor, stepper motor controller, ESP32 microprocessor, and sensor controller breakout boards. The ToF sensor, stepper motor controller, and ESP32 microprocessor breakout boards are commercially available; the pressure sensor and sensor controller boards are custom-made. To construct the pressure sensor breakout board, the surface mount pressure sensor (*illustrated) is first soldered with headers onto a small through-hole mount board, and then this carrier board is soldered onto a circuit board. The supplementary information details how this and the sensor controller breakout board are constructed. Image attribution: **Adafruit. (**b**) Annotated LungElast instrument. The location of the electronic components and cable connections in an assembled LungElast is shown; the above numbers identify each part. The ribbon cables facilitate the I^2^C digital communications, and the twisted wires power the stepper motor coils. Scale: 25-cent coin.

The electronic components of LungElast and their electrical connection with the sensors and stepper motor are shown in Fig. 3. LungElast employs sensor-on-chip technologies to provide digital airway pressure and syringe positioning information to its microcontroller via a serial inter-integrated circuit digital interface communications data bus (I^2^C). While maintaining data reliability, this design feature eliminates the need for analog pressure and linear transducer sensors, signal conditioners, ADC devices, and chart recorders, which have been used in other elastometers^13,38^. LungElast uses a gauge pressure sensor incorporating an ADC subunit with a 16-bit digital resolution and an I^2^C interface generator on the chip. The device specified in this build (All Sensors, DLC-L20G-U2) is optimized to measure pressures in the 0–51 cm H_2_O range. But if an instrument with a different range is desired, the manufacturer produces other sensors with the same footprint that might meet the need. This sensor’s low power consumption and supply voltage requirements mean it can be powered directly by the device microcontroller.

To enable the automatic function, an elastometer controller needs to be able to move the piston to a position where the syringe is full of air and to ensure that the instrument’s motor does not drive the piston beyond the instrument's limits. The LungElast microcontroller acquires piston position information through the novel use of a micro time-of-flight light detection and ranging proximity sensor (ToF, STMicroelectronics, VL6180). Data from this sensor allows the microprocessor to instruct the stepper motor to initially move the piston to a home position so the syringe is nearly full of gas. Then, positioning data are acquired from the sensor while the piston is being moved to introduce and remove gas from the lung so that the microcontroller does not instruct the motor to drive the piston beyond the syringe's maximum and minimum barrel limits. The ToF sensor used by LungElast provides range information by emitting IR photons that bounce off the surface of a target incorporated in the lead screw nut that holds the syringe piston flange. After capturing and determining the transit time of returning photons, the sensor calculates the absolute distance between the sensor and the target. The contactless nature of the syringe piston position determination by the ToF sensor eliminates eccentric and drag forces on the piston control unit. Although micro ToF devices are increasingly being incorporated into smart devices to aid in gesture sensing, this is the first use of this type of sensor that I am aware of in syringe pumps.

LungElast is controlled using the multifunctional ESP32-WROOM-32 32-bit, dual-core, RISC-V, 160 MHz microprocessor. This low-power microprocessor employs a system-on-chip design with integral hardware timers, general-purpose input and output (GPIO) pins, and successive approximation register-type ADC subunits^45^. In LungElast, the hardware timers are used to precisely control the stepper motor movements and timing of pressure measurements. The GPIO pins are used to manage the power to the LungElast sensors; the ADC is used to determine the voltage level of the device power supply. The ESP32 microprocessor also supports LungElast’s high degree of connectivity. The microprocessor has modules that control I^2^C, universal asynchronous receiver-transmitter (UART), and 802.11b/g/n HT40 Wi-Fi communications. The I^2^C bus is used for data transfer between the microprocessor, stepper motor controller, and sensors, as graphically illustrated in Fig. 1c. The UART communications are used during the initial upload of the MicroPython interpreter and LungElast scripts onto the device. Subsequently, the user can employ wireless communications to control the device and obtain desired data. Notably, the ESP32 microprocessor is available in an inexpensive breakout board with other important features. The breakout board has circuitry that facilitates USB communications and lithium polymer (LiPo) battery use. The breakout board can also snap into a carrier board, making power and signal interconnections between the ESP32 and the other LungElast breakout and control boards. Images of the LungElast breakout boards are shown in Fig. 3a. Three of the LungElast boards are commercially available; two require custom construction. The online materials provide detailed wiring diagrams, assembly instructions for the later boards, and a LungElast schematic. The sources and current prices of the components are listed in the *Bill of Materials.*

### Software design

The LungElast software was designed to enhance the device’s flexibility and ease of use. MicroPython is used to interpret the device’s scripts because it is optimized to operate efficiently in memory-constrained microprocessors, such as the ESP32. The LungElast software consists of a suite of tools. This approach gives the user the flexibility to employ the individual scripts, as needed, to test the hardware, calibrate the sensors, define and save the device control parameters, and automatically obtain lung mechanics data. A list of the scripts and their utilities is shown in Table S2. The software is provided online in MicroPython precompiled bytecode code (mpy) format, which decreases its memory requirements and enhances its performance, and compilable text file (py) format.

**Figure 4:**
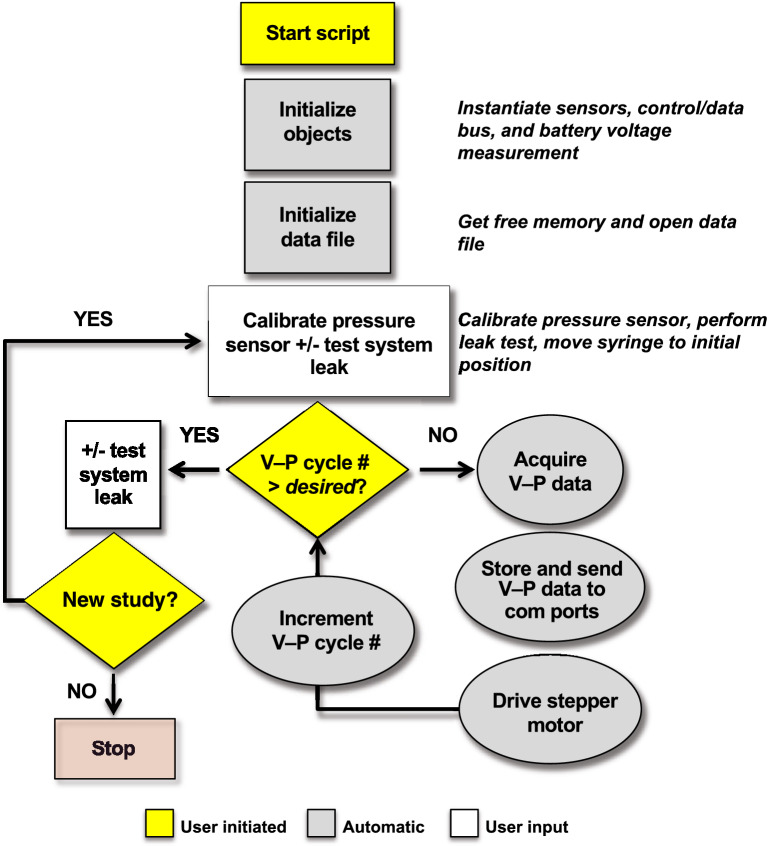
Flow diagram of the LungElast.mpy script. After a communications link is established with the LungElast device, the script is started. Then the script initializes the sensors and loads into memory the device control and experimental parameters that were stored in the customizations.json. This file is previously generated by the user employing Customizer.mpy. Then, the user is prompted to calibrate the pressure sensor using an attached manometer and to test the gas system for leaks. Subsequently, the instrument automatically advances the syringe plunger—injecting previously determined volumes of air into the attached airways—obtains pressure measurements and reports and records the V-P data. After the desired predetermined number of V-P cycles is completed, the data collection terminates. The user is then given the option to test the system and lung for leaks. Finally, the user is given the opportunity to perform additional V-P experiments or to stop the script. The user can then download the data and experimental parameters from the device.

LungElast.mpy is the main script that drives automated V-P data acquisition by the device. Its flow diagram is shown in Fig. 4; video demonstrations of the use of this and the scripts described below are in the online supplementary information. During V-P data collection, LungElast.mpy tallies the motor step counts and uses the volume calibration factor to derive the accumulated lung volumes based on the syringe piston position. It also compares the current airway pressure measurement values with the user-defined maximum and minimum pressure thresholds to automatically drive the syringe piston to inject or remove lung gas. This eliminates the need for the user to manually switch the direction of the syringe piston movement during the experiment. The software also introduces a short pause between the motor steps to compensate for the inertia of the piston-actuating system. Another delay is introduced between the train of motor steps to allow the gas to distribute throughout the lung and stabilize before pressure measurements are made. Because the optimal value for these timings depends on the lung's size and time constants, the user can define them using the LungElast parameter customization script described below.

As a design feature that enhances automation, LungElast.mpy also regulates the piston direction movement based on the ToF-determined syringe piston position and the maximum and minimum piston excursion limits set by the user. The script also allows the user to automatically change the delivered or removed gas volume increments near key pressure targets. This feature increases the number of V-P data points available to the user to aid in defining fundamental physiologic values (e.g., elastance, TLC, RV). Also, LungElast.mpy allows the user to detect leaks by determining the change in system pressure leak over time. The software prompts the user to apply a set pressure to the gas-conducting system without or with the lung connected in continuity to it. Then the software measures the pressure levels over time, calculates the rate of pressure change, and reports the information to the user. This feature helps the user test the integrity of the ventilation circuit connections and determines whether the lung developed a gas leak during an experiment. Lastly, LungElast.mpy directly saves V-P data in the nonvolatile microprocessor memory to eliminate the need for a chart recorder. These data are indexed with experiment date and subject information and can be downloaded from the device using the hard-wired or Wi-Fi communications connection.

Other scripts are provided to aid the user in calibrating the hardware system and storing the parameters used to control the device during the experiment. For example, DistVolCal.mpy enables the builder to use the ToF sensor to identify acceptable piston plunger travel distances for the device and to determine how much volume the syringe piston delivers under the control of the stepper motor. Its flow diagram is shown in Figure S2. CompCal.mpy can be employed by the user to determine the pressure-dependent compression of gas volume delivered by the LungElast device during a V-P experiment. A discussion of the utility of this compression factor is described elsewhere^13^; the method by which the CompCal script helps the user determine this factor for their LungElast build is described in Table S2. LungElast automatically determines and reports the corrected volume measurements during a V-P experiment if the volume compression factor is determined and saved in the parameters. The Customizer.mpy script enhances the flexibility of LungElast. It allows the user to save the calibration data described above and define the parameters that the LungElast.mpy employs during the V-P experiments. It also permits the user to specify the LungElast stepper motor movement and timing, V-P sampling, piston position parameters, pressure calibration and target values, and other experimental parameters defined in Table S3. Customizer.mpy can also be employed by the user to export the experiment control parameters for documentation. For example, Table S4 shows the experimental parameters used during the lung validation experiments described below.

The rate at which LungElast moves gas in the lung should be chosen based on the experimental aims, tempered by the size of the mouse. Its rate capability is a function of the hardware used to make the device and the motor control parameters. The choice of the stepper motor, lead screw, and syringe dimensions plays an important role, including the number of motor steps per lead screw rotation, the pitch of the screw teeth, and the diameter of the syringe piston. The motor step rate, gas pressure settling time, and the frequency of pressure and piston position measurements set by the user play a role in controlling the rate. In adult mouse studies, others used a gas movement rate of 3 ml/min so that experiments are not so short as to allow dynamic tissue elasticity factors to affect the results and not too long to be technically impractical^13^. This rate can be achieved with LungElast using the build described here. Driving LungElast built with a 2 mm pitch lead screw and a 3 ml syringe with a 9 mm diameter piston at a motor rate of 1 step per 10 µs can move gas at ~ 4 ml/min rate. In mouse pup lung elasticity experiments, such as the ones performed below, a syringe with a smaller diameter piston was constructed, and a lower motor drive rate was used (specified in Table S4).

### Validation of sensors and volume delivery

Surprisingly, few data are available detailing the linearity, precision, and accuracy of the pressure measurements and volume delivery of previously described elastometers. Because this information is critical for determining the robustness of such instruments, I determined these values using LungElast over a range of volumes and pressures used to study mouse lung mechanics.

#### Validation pressure measurement

**Figure 5:**
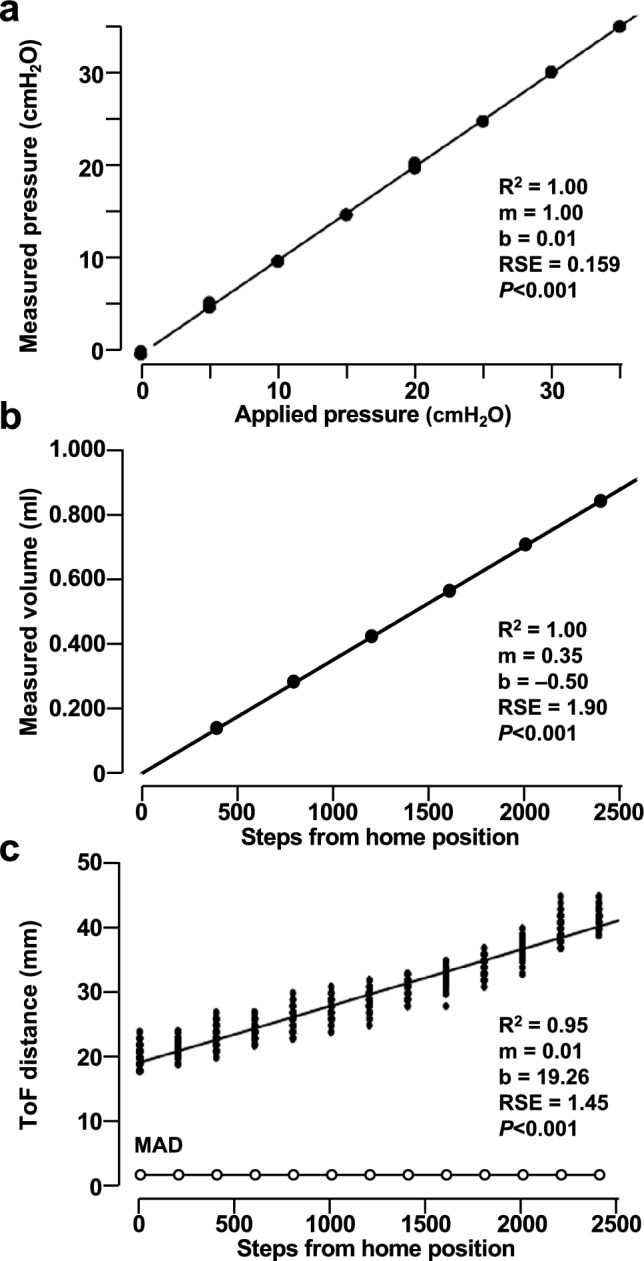
LungElast sensor and volume delivery validations. (**a**) The DLC-L20G-U2 sensor detects pressure with high accuracy and low variance in a linear manner. After applying the indicated gas pressures to the device, seven single-shot pressure measurements were obtained using the LungElast digital pressure sensor. (**b**) LungElast reproducibly delivers volume in a linear manner with low variance. In 7 separate experiments, the volume expressed by a 1 ml water-filled syringe whose piston was advanced by a 2 mm pitch lead screw rotated the indicated number of full stepper motor steps was collected and quantified using gravimetric methods. (**c**) The VL6180 ToF sensor detects the LungElast syringe piston in a linear manner. After moving the lead nut target by rotating the lead screw the indicated number of full stepper motor steps, 200 single-shot range measurements were obtained using the sensor. Closed symbols: individual data points, linear regression model line, and associated statistics are shown; in (**a**) and (**b**), the low data variance did not permit the individual data points to be resolved. Open symbols: median absolute deviation (MAD) of the ToF distance measurements. The goodness of fit (R^2^), slope (m), and intercept (**b**) of a linear model, residual standard error (RSE), and significance of the *P* value for the estimates are shown.

First, I tested how well the DLC-L20G-U2 sensor measures pressures. As shown in Fig. 5a, this sensor was determined to produce linear data over the relevant pressure range with low variance and high accuracy. The repeated, single-shot pressure measurement values clustered closely with each other and correlated highly with the applied pressure. I next determined how many pressure measurements would be necessary to yield data of a specified accuracy. Analysis of the pooled pressure measurement data deviation across the pressure range using the z-score estimator determined that seven sequential single-shot measurements would be necessary to yield pressure data within 0.1 cm H_2_O of the expected values with 95% certainty. As a result, LungElast.mpy acquires seven pressure measurements during V-P data collection.

#### Validation of volume delivery

I next tested whether the LungElast stepper motor drives precise and reproducible volume delivery. This experiment tested the correlation between full motor steps and volume deliveries using water instead of gas because it is non-compressible and can be accurately quantified using gravimetric methods. Analysis of data collected during seven independent experiments determined that LungElast delivers small volumes in a reproducible and linear manner with low variance (Fig. 5b).

#### Validation of syringe piston positioning using a time-of-flight sensor

LungElast uses a VL6180 micro ToF sensor to determine the lead screw position of the nut–syringe piston flange assembly. I next acquired data to determine the precision and resolution of its measurement of plunger distances corresponding to where the syringe is filled with air (home position) and where it is nearly emptied. As shown in Fig. 5c, the relationship between motor steps and ToF-determined syringe flange distances was linear. But these data exhibited a higher variance than those determined during the pressure and volume delivery validation measurements. Nevertheless, the data indicate that the ToF sensor provides sufficient position precision for LungElast to regulate the syringe pistons; the median absolute deviation was only ~ 1.5-mm across the distance measurement range. Analysis of the pooled ToF distance measurement deviation using the z-score estimator determined that five single-shot ToF range measurements would result in distance measurements within 1.5-mm of the expected values with 95% certainty. As a result, the LungElast scripts are programmed to make five range measurements during the syringe piston positioning.

### Validation of V-P measurements using elastomeric lungs

Because LungElast determines the pressure that results from a change in lung volume, I use the data generated in the validation experiments described below to construct V-P graphs and derive elastance measurements. I supply P–V curves and compliance measurements in the online supplementary information because these data representations are also shown in the literature.

To validate the acquisition of V-P data by LungElast, I constructed two elastomeric lung models: one with relatively thin latex and another with a thicker latex wall. Details about their construction are provided in the online supplementary information. The LungElast control parameters were set using Customizer.mpy so that the instrument would automatically decrease the volume changes, thereby increasing the frequency of pressure determinations near the maximum and minimum target pressures for the lung models. Table S4 documents the control parameters used in this experiment. For the thin wall model, 40 µl gas was sequentially injected until the pressure was within 30% of the maximum target, then LungElast automatically started injecting only 10 µl of gas each time. During the deflation, 40 µl of gas was removed with each step until within 30% of the minimum target pressure, at which point only 20 µl of gas was automatically withdrawn at each step. For the thick wall lung model, in which volume injection and withdrawal cause greater pressure changes, 20 µl gas was sequentially injected until 30% of the maximum target pressure. Then, increments of 10 µl of gas were injected until the maximum target pressure was reached. During deflation, 20 µl gas was sequentially withdrawn. When within 30% of the minimum pressure, LungElast automatically withdrew 10 µl of gas sequentially. For the mouse pup lungs, 40 µl of gas was injected or removed throughout the V-P experiment.

**Figure 6:**
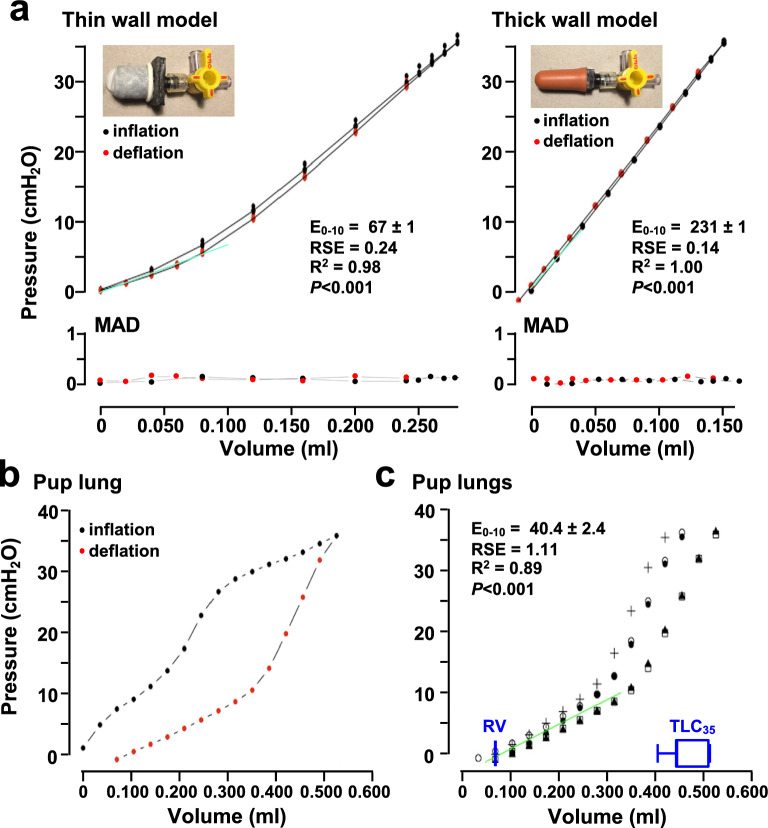
Lung V-P measurements. (**a**) V-P data from elastomeric lung models. The pressures resulting from LungElast sequentially injecting the indicated accumulated gas volumes (closed black symbols, inflation) into lung models with thin (left) and thick (right) latex walls were determined. After a pressure of 35 cm H_2_O was achieved, the device automatically started removing gas volumes (closed red symbols, deflation) until atmospheric pressure was reached while pressures were measured. The individual measurements from 7 separate V-P experiments are shown, with the black lines connecting average values. The variability of the V-P results is too small to allow the individual data points to be discerned. The V-P relationships at deflation pressures ≤ 10 cm H_2_O were modeled using linear regression (green line), and the change in pressure associated with volume yielded the elastance (E_0–10_, in cm H_2_O/ml); shown are the estimates ± standard error, RSE, R^2^, and significance of the *P* value. The MAD of the pressure measurements during the air inflation and deflation are also shown with the expanded scale. (**b**) V-P data from mouse pup lungs. The pressures that resulted from sequentially injecting the indicated volumes of air into the lungs of a P21 mouse until a pressure of 35 cm H_2_O was achieved and then removing the same volumes of gas until 0 cm H_2_O was detected. (**c**) The deflation V-P curves of 5 P21 mouse pups were studied similarly as in (**b**); each symbol represents data derived from a mouse pup. The box plots (blue) represent the residual volume (RV) and total lung capacity at 35 cm H_2_O inflation pressure (TLC_35_) of the mouse pups, as indicated.

Seven independent inflation and deflation cycles were performed for the validation work using each lung model. The individual volume and pressure data points obtained during the experiments are shown graphically in Fig. 6a. Moreover, the elastance of the model lungs was calculated using linear modeling of the V-P values for pressure values < 10 cm H_2_O during the deflation limb of the curve. Because, similar to what is observed in mouse lung studies (^20^ and below), the change in pressure and volume in this pressure range is observed to be constant. The experiments with the elastomeric lung models indicate that LungElast produces highly reproducible V-P data. Across the entire volume range, the pressure values had low variance; the median absolute deviation was < 0.2 cm H_2_O pressure. Moreover, and as expected, the thin-walled lung model exhibited a lower change in pressure with each volume increment compared to the change observed with the thicker-walled one. Although pressure is the dependent variable, the data shown in Fig. 6 are sometimes represented in a P–V format, with the pressures labeled on the abscissa and the independent volumes on the ordinate axis. Accordingly, a representation of the same data in this format, and compliance determinations, is shown in Supplementary Figure S3.

### Feasibility of determining pulmonary mechanics using young mice

Lung V-P data were obtained using LungElast constructed with a 1 ml syringe and five mouse pups from the same litter without regard to their sex. Pups that were 21 days old (P21) were used in this experiment. Their lung extracellular elastin and alveolar development are nearly complete by that age^46–48^. As a result, I expected that their lungs might yield full-featured V-P curves. These immature mice were also used to demonstrate the feasibility of using LungElast to measure lung elastance, even in small animals. The weight of the pups was 8.5 ± 1.0 g. After a mouse pup was euthanized and its chest was opened to allow the lungs to collapse to residual volume, a blunt needle was secured in the trachea. The pup was connected to LungElast, as shown in Figure S1. After initiating the LungElast script, the pressure sensor was calibrated using a water manometer. Then V-P data were acquired with the lungs in situ. Although the TLC of mouse pups has not yet been established, a maximum target pressure of 35 cm H_2_O was used in this experiment. Other investigators have used this threshold pressure in adult mouse studies^13,30^.

As shown in Fig. 6b, a graphical representation of the V-P data of a typical mouse pup exhibited several features that have been observed in adult mice^22,30,49^. This includes a sigmoidal relationship between the volume and airway pressures during the experiment's lung inflation and deflation cycles. Moreover, a concavity was detected in the inflation data graph that others report might reflect lung volumes during which airways are recruited^20^. The curve of the airway inflation data also did not appear to be asymptotic, which is similar to what has been detected in adult mice^30^. As expected, hysteresis in the inflation and deflation data was also detected, which was not observed in the elastomeric lung models. The source of this hysteresis has yet to be fully understood, though it has also been seen in the studies of immature and mature lungs from various species, including mice^13,19,50–53^. The deflation curve exhibited a linear V-P relationship at pressures ≤ 10 cm H_2_O, which is similar to what was observed by others^20^. This linear range permits the data's modeling and the determination of lung elastance.

The reproducibility of the V-P measurements is demonstrated by inspecting the deflation curves of the mouse pups shown in Fig. 6c. Linear modeling of the deflation data between 0 and 10 cm H_2_O pressure yielded the elastance of the lungs (E_0–10_). It revealed low variability in the pup lung stiffness, as evidenced by a low residual standard error detected during the linear modeling (Fig. 6c). Although elastance has not previously been reported for P21 mouse pups, the values obtained by LungElast are lower than those detected in P14 mouse pups, obtained using a ventilator system^25–27^, and slightly higher than those reported in adult mouse lungs, obtained using syringe pump^13,20,38^ and ventilator systems^38,54^. These results are expected because previous studies show that elastogenesis is more complete in P21 pups than in younger ones^10,46,55^, and that lung elastance decreases even after that age^7^. The lung volume at an inflation pressure of 35 cm H_2_O (TLC_35_) was 0.476 ± 0.046 ml, and the lung volume at a deflation pressure of 0 cm H_2_O (RV) was 0.070 ± 0.025 ml. The P–V representation of these same mouse pup lung data, and compliance measurements, are shown in Supplementary Figure S4.

### Limitations

One limitation of LungElast is that a certain level of technical ability is required to manufacture the parts, make pressure sensor breakout and control boards, and assemble the device. Moreover, some knowledge of microprocessors is necessary to flash the MicroPython interpreter and transfer the LungElast scripts to the device. But detailed instructions are provided, and a moderately proficient maker can likely produce the instrument. Because it is so inexpensive, the maker could construct several LungElast devices simultaneously for parallel experiments or share them with other laboratories to offset the effort. Another limitation of the device is that LungElast is not designed to acquire continuous V-P data. Although the ESP32 computer has two microprocessor cores that could facilitate the simultaneous control of gas volume delivery and measurement of airway pressures, the MicroPython interpreter allows access to only one. But given that this interpreter is constantly being developed and upgraded, its later modifications might make these capabilities available. In addition, like most syringe pump elastometers, LungElast cannot assess the effects of positive versus negative pressure ventilation on the same specimen or measure lung viscoelasticity. If these measurements are desired, other devices might be built or purchased to obtain this information.

## Summary

LungElast is presented as an open-source, adaptable, scalable, and inexpensive instrument that can automatically measure the mechanical properties of mouse lungs. The strengths of this device include its microprocessor control, which significantly enhances the flexibility of the device. In addition, vital mechanical portions of the device are designed to be scalable. Accordingly, LungElast can be modified to utilize syringe pistons of various sizes. This could extend its use for determining lung mechanics in mice and other small mammals of varying sizes.

## Materials and methods

### LungElast fabrication

To construct LungElast, the online components listed in the Bill of Materials were acquired, and the detailed build instructions were followed^17^. After slicing the 3D modeling Standard Tessellation Language (STL) files, the custom mechanical parts shown in Fig. 2 were made using a fused deposition modeling 3D printer (Prusa Research, i3 MK3S +) and polylactic acid (PLA) filament. The device was then assembled in the following manner. The lead screw was rotated so that ~ 10 cm protruded from the working end of the motor. The stepper motor was then oriented so that its wires exited its body upward, and then it was attached to its holder using the lower two screws. Care was exercised not to over-tighten these or the other screws in the construction. Then, two screws were introduced through the upper ToF holder and the upper screw mounts of the motor holder and then secured in the upper mounting holes of the stepper motor. The ToF breakout board was screwed into its holder, so its header is oriented upward. While the syringe barrel laid its rest, its flange was captured by the syringe barrel flange lock, which was then secured using two bolts. Then, the syringe plunger flange was slipped into the lead screw nut. The syringe piston was lightly lubricated using silicone paste and introduced into the barrel. With the nut oriented so that the ToF target was upright, the lead screw guides were snapped onto the sides of the syringe body rest and stepper motor holder while capturing the wings of the nut. The lead screw was manually turned until it exited the motor and entered the nut. The Luer lock connectors and manometer used for the pressure calibration were assembled as shown in the diagram in Fig. 1. The sensor controller and pressure sensor breakout boards and ribbon cables were constructed according to the schematics and detailed in the online supplementary information. The stepper motor, ESP32 microcontroller, and sensor controller boards were then snapped into an interconnector board (i.e., Adafruit, FeatherWing Tripler Mini Kit). The ribbon cables were then connected to the breakout boards as the online supplementary information described.

The next step involved installing the MicroPython interpreter onto the ESP32 microprocessor. I followed the information in the extensive online tutorials referenced in the supplementary materials. Briefly, a software driver for the USB to TTL converter device (SiLabs, CP2104), ESP32 memory flashing tool (Espressif, esptool.py), MicroPython interpreter (MicroPython.org, version 1.9), and shell program (e.g., ampy) were downloaded onto a host PC from online repositories. Then, after establishing a serial connection between the host computer and the ESP32 breakout board using a data USB cable, esptool.py was used to load the interpreter into the microprocessor’s flash memory. At that point, the installation was confirmed by entering the MicroPython REPL using the shell program. Alternatively, some builders prefer to use a MicroPython integrated development environment program (e.g., Thonny) to accomplish this work. Subsequently, the LungElast scripts and required libraries were loaded from the online repository onto the ESP32 microprocessor using the shell program. These scripts are detailed in Table S2.

Finally, the instrument was tested and calibrated in the following manner. The power connection to the ESP32 microprocessor breakout board from the USB cable was confirmed by noting a yellow light emitting diode (LED) flashing on it; the power to the stepper motor control board was also confirmed by observing a LED illuminated on it. The I^2^C and power to the sensor and controller breakout boards were tested using BusTester.mpy. Next, the syringe piston positioning and delivery volumes were determined using the above scripts. Finally, Customizer.mpy was used to update the calibration parameters obtained during the previous steps in the customizations.json file, which LungElast uses to get V-P data.

### Pressure sensor validation

The relationship between applied and measured pressure levels was determined in the following manner. The stopcock that connects LungElast with the animal subjects was closed (Fig. 1), and the airtightness of the system was confirmed. Then air was introduced into the LungElast system using a gas-tight syringe connected to a stopcock to generate increasing pressure levels in the device as measured by the manometer. Seven calibrated gauge pressure measurements were obtained at each level using the single-shot sensor. The data were then analyzed as described below.

### Volume delivery validation

The linearity and precision of volumes delivered by LungElast were determined in the following manner. A blunt needle (27 g) was attached to a short length of polyethylene tubing (Stoelting, PE20) and then secured on the LungElast syringe Luer connector. Then DistVolCal.py was entered into the volume calibration mode and employed to empty the syringe of air, fill it with water, and purge residual air. Then volumes of water extruded by the device were collected in pre-weighed microcentrifuge tubes while the lead screw advanced the syringe plunger during a series of 400 full motor steps. The volume of water in each tube was then determined gravimetrically using a calibrated and protected scale (Mettler, AB104). A range of volumes up to ~ 900 µl was studied because a pilot study suggested that the TLC_35_ of the mouse pups used in this study is approximately 500 µl. This experiment was repeated seven times.

### Time-of-flight light detection and range determination validation

Studies were performed to test the ToF sensor linearity and precision and to define the number of range samples needed to determine the syringe piston position. ToF sensor averaging sampling period (ASP), ambient temperature, and ambient light level influence the validity of range determinations. Increasing the ASP decreases sensor noise at the expense of growing range determination time and sensor power consumption. An ASP of 13 ms was used in this and subsequent work because pilot studies suggested that it was the most prolonged period that first yielded decreased range data variance in our system. Each experiment was conducted in subdued light after an automated sensor temperature calibration. Two hundred single-shot ToF range measurements were obtained at each of the 13-lead nut screw target positions to test the linearity and precision of the ToF range measurements. The range of these positions corresponds with the excursion limits of the syringe plunger used in a mouse lung elastance experiment. The range measurements described above were analyzed to determine the optimal number of ToF range samples resulting in plunger position estimates with the below-mentioned accuracy level.

### Validation of utility in obtaining V-P data

The efficacy of acquiring V-P data and determining lung elastance using LungElast was tested using elastomeric models and mouse pup lungs. For the studies using the models, elastomeric latex lungs with two wall thickness levels were constructed using commonly available materials, 3D printed mounts, and methods detailed in the online supplementary information. The mouse pup experiments were approved by the Institutional Animal Care and Use Committee (IACUC) for MGH and Shriner’s Hospital, performed following relevant guidelines and regulations, and reported using ARRIVE guidelines. In preparation for the experiment, postnatal day 21 (P21) C57BL/6Ncr mouse pups were euthanized by intraperitoneal injection of sodium pentobarbital (20 mg/kg body wt.). Then the lungs were accessed as described previously^56,57^. Briefly, the abdomen was opened, and a hole was made in the diaphragm to permit the lungs to collapse. Although previous studies using adult mice indicate that the thoracic wall plays a minor role in lung mechanics^20,33^, its effect on those with the developing lung is unknown. Accordingly, the anterior chest wall was dissected away from the lungs for this study. A blunt needle (21 g) was introduced into the trachea through a small incision made with iris scissors and secured using silk ligatures and cyanoacrylate (Loctite, 1,363,589). Then the mouse pup was connected to LungElast, as shown in Figure S1. The V-P relationships during a single inflation and deflation cycle were determined using LungElast, and the experimental parameters shown in Table S4.

### Data analysis and statistical methods

The data were analyzed using R^58^. Unless otherwise noted, all data are represented as mean ± SD. Linear regression was used to determine the relationship between the independent and dependent data parameters. The slope and intercept of the linear model, the residual standard error (RSE), the goodness of fit, and the significance of the *P*-value for the estimates were determined. In some cases, the median absolute deviation of the data was also calculated. The number of sensor samples (n) required to yield estimates within the specified range (E) with 95% certainty was determined using the z-score equation: n = (z_0.05_ × σ/E)^2^, where σ was the pooled standard deviation. The elastance and compliance values were determined by fitting the elastomeric model and mouse pup lung deflation data, within the specified pressure range, to a linear model using regression.

## Supplementary Information

Supplementary Information 1.

## Data Availability

The data are available from Jesse D. Roberts Jr. upon request.
