# Publisher Correction: LungElast—an open-source, flexible, low-cost, microprocessor-controlled mouse lung elastometer

**DOI:** 10.1038/s41598-023-39549-w

**Published:** 2023-08-01

**Authors:** Jesse D. Roberts

**Affiliations:** 1 Cardiovascular Research Center of the General Medical Services and the Departments of Anesthesia, Critical Care and Pain Medicine, Pediatrics, and Medicine, Massachusetts General Hospital - East, 149 13th St, Boston, MA USA; 2 Harvard Medical School, Harvard University, Cambridge, MA USA

Correction to: *Scientific Reports* 10.1038/s41598-023-38310-7, published online 12 July 2023

The original version of this Article contained an error in the name of author Jesse D. Roberts Jr., where the name suffix ‘Jr.’ was inadvertently omitted.

The original Article has been corrected.

