# Supplementary material for: LungElast—an open-source, flexible, low-cost, microprocessor-controlled mouse lung elastometer
Source: Sci Rep. 2023 Jul 12;13:11246. doi: 10.1038/s41598-023-38310-7 (PMC10338507; doi:10.1038/s41598-023-38310-7)
Supplement: Supplementary file 1 — Supplementary Information 1. [file 41598_2023_38310_MOESM1_ESM.pdf]

Index of the online Supplementary Information. For this manuscript review, the files that are provided online are also supplied in following pages. But please note that the LungElast MicroPython script and hardware stl files are in the OSF and TinkerCad repositories and not supplied below. URL links below for that material are below.

Supplementary Information	SI page
Fig. S1	2
Fig. S2	3
Fig. S3	4
Fig. S4	5
Table S1	6
Table S2	8
Table S3	10
Table S4	12
Bill of Materials	14
Elastomeric model lung construction	17
ESP32 and MicroPython resources	20
Pressure sensor breakout board construction	22
Pressure sensor breakout board wiring	27
LungElast cables	29
Sensor controller board wiring	30
Schematic	32

OSF: https://osf.io/32ury/?view_only=de948fde5bf9431d81780894cc87f4e1

TinkerCad: https://www.tinkercad.com/things/gCcWHAqKk7O-lungelast-3d-models/edit?sharecode=1FjriaGIr2fiWI3po5UaQyUdvunhnkCfJw3noIZeQTE

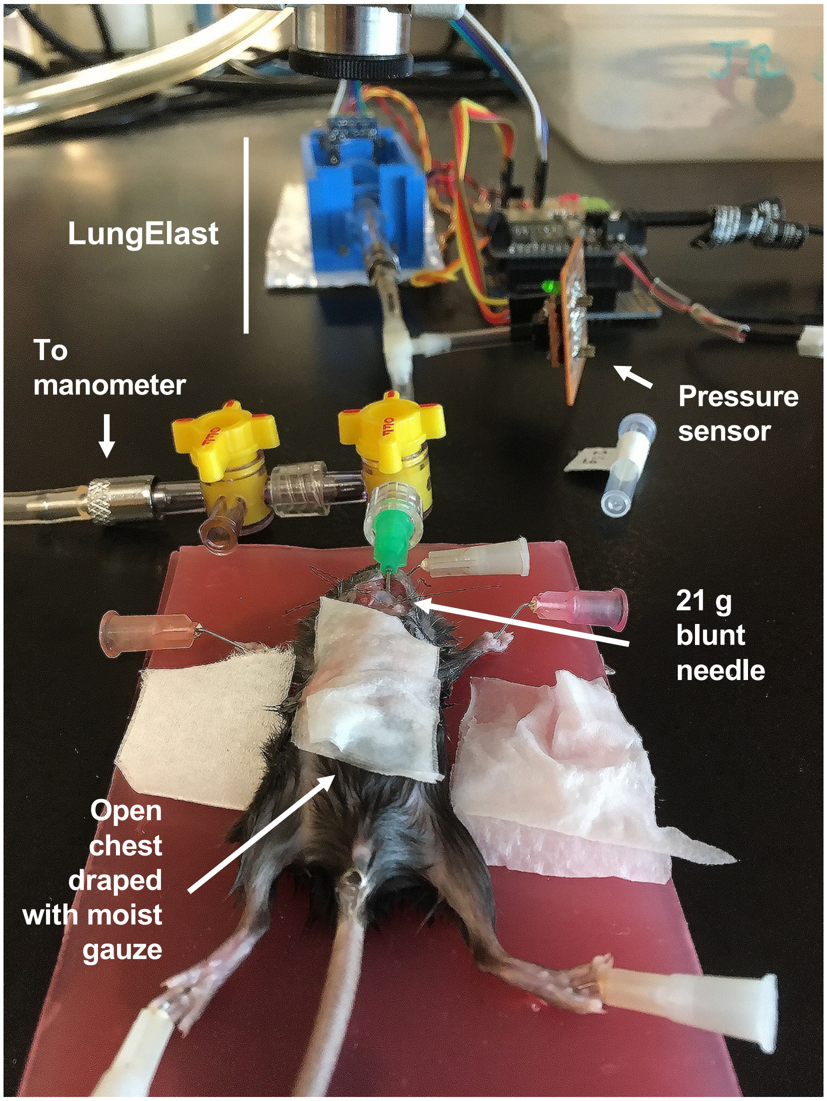

Figure S1. P21 mouse pup elastance experiment. This figure shows the laboratory bench setup of a V-P experiment using a P21 mouse pup and LungElast. The details of the animal preparation are in the *Methods* section of the manuscript. A 21 g blunt needle is secured in the mouse pup trachea; the chest is opened so the lungs can collapse to residual volume; moist gauze is draped over the thorax so the lung surface does not dry. The blunt needle is connected via tubing and stop-cocks to LungElast and a manometer. At this point, at the beginning of the experiment, the stopcocks are arranged so that LungElast is connected to the manometer so that its pressure sensor can be calibrated.

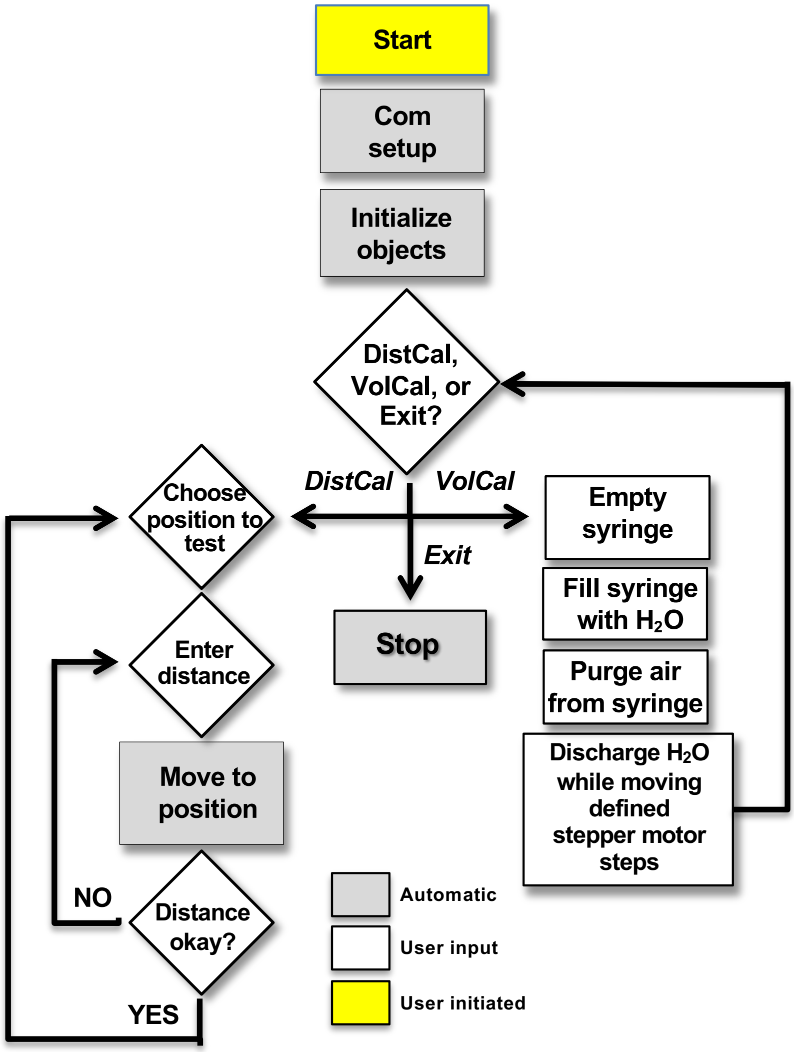

Figure S2. Flow diagram of the DistVolCal script. After a communications link with LungElast is established and it initializes the sensor objects, the user is given the option to calibrate the syringe plunger movements, its volume delivery, or to stop the script. For the position calibration, the user enters estimates of the maximum and minimum lead nut - ToF sensor target positions for the system that they constructed. The 5mm markers printed on one of the lead nut guides can aid in these measurements. Then, the script instructs LungElast to move the syringe plunger to the indicated distances and asks the user to confirm that the positions are satisfactory or whether they need to be revised. For the volume delivery calibration, the syringe is emptied by its plunger being moved to the maximum location set above. The user is then asked to attach flexible tubing to the syringe and immerse its free end in water. Then the syringe plunger is moved to the minimum location while filling with water. After the device purges air remaining in the syringe, the user is asked to move the free end of the tubing to an empty receptacle and then the motor moves a predetermined number of steps and empties water into the container. After the user determines the extruded volume gravimetrically, they can calculate the volume delivery. The calibrated positions and volume delivery results are then updated for the device using the customizations script. The user can the repeat these calibrations or stop the DistVolCal script.

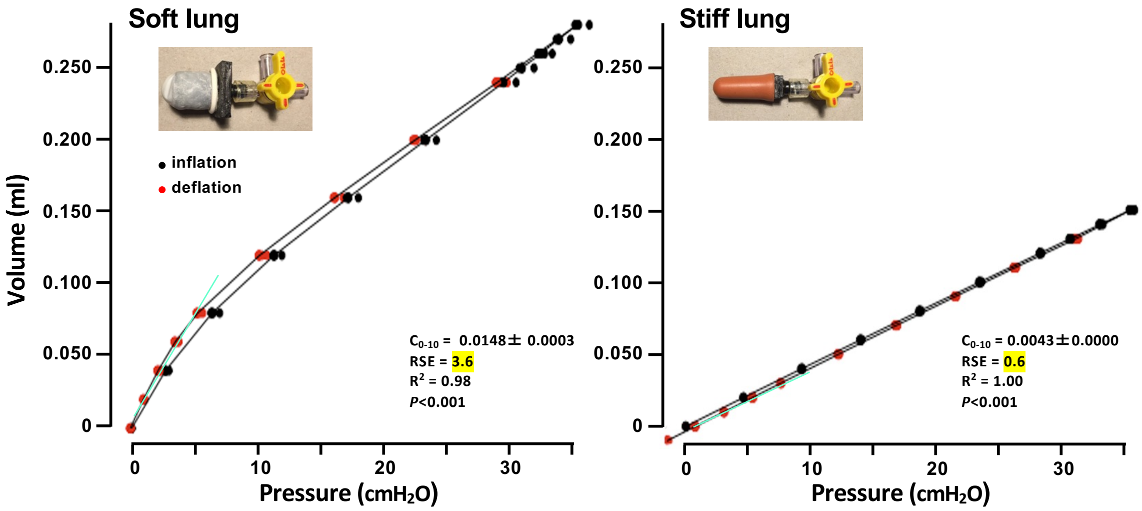

Figure S3. P-V validation data using elastomeric lungs. The pressures resulting from LungElast sequentially injected volumes of gas (black circles) into latex model lungs with soft (left) and stiff (right) elasticity until a maximum pressure of 35 cmH_2O was quantified. Subsequently, the pressures resulting from removing volumes of air (red circles) were measured until atmospheric pressure was achieved. The individual measurements from seven separate experiments are shown, with the lines connecting average values. In most cases, the variability in the individual P-V results was too small to allow the individual data points to be discerned. The P-V relationships for deflation pressures ≤ 10 cmH_2O were modeled using linear regression (green line) and the change in pressure associated with volume was calculate yielding the model lung elastance (C_{0-10}, in ml/cmH_2O). The residual standard error (RSE), R-squared, and *P*-value are shown.

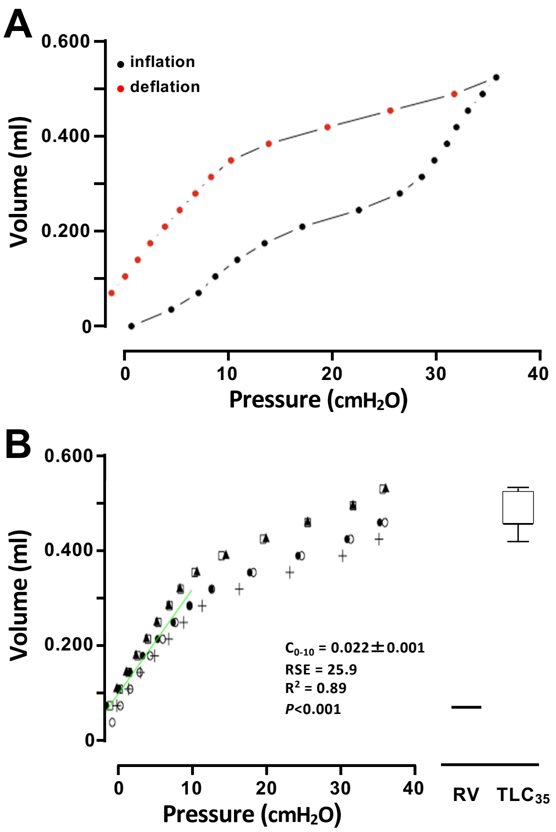

Figure S4. P-V validation data using P21 mouse pup lungs. *A.* The pressures resulting from sequentially injecting 35 µl volumes of air until a pressure of 35 cmH_2O was detected (black circles) and then incrementally removing the same volume of gas (red circles) from a representative P21 mouse lung in situ. ***B.*** The deflation P-V curves of 5 P21 mouse pups; each symbol represents data derived from a different mouse pup. The P-V relationships for deflation pressures ≤ 10 cmH_2O were modeled using linear regression (green line), the slope yielding the compliance ($C_{0\text{-}10}$, ml/cmH_2O); shown are the estimate ± the standard error. The R^2, *P*-value, and residual standard error (RSE) are shown. The box plots represent the residual volume (RV) and total lung capacity (TLC_{35}) of the 5 mouse pups, as indicated.

Table S1: Common lung mechanics parameters extracted from V-P data.

Parameter	Definition
Total lung capacity (TLC or TLC_x, where x is the pressure chosen to define TLC)	The lung volume associated with its maximum inflation. Data suggest that adult mouse lung does not exhibit a plateau that is indicative of maximum inflation during the inspiratory portion of the V-P curve [1]. Moreover, the airway pressure that corresponds with maximum lung volume appears to vary with the strain of the mouse [2].
Residual volume (RV)	The residual air trapped in the distal lung structures at maximum expiration. During V-P studies, this represents the volume of gas residing in the lung at the end of the first deflation curve [3].
Indexed lung volume (%V_x, where x is the lung volume)	The % lung volume at x cmH_2O pressure. Johnson and coworkers determined that %V_{15} correlates with the minimum surface tension in adult dogs [4]**.** Later studies indicated that %V_{10} correlates with an improvement in alveolar stability in adult mice [5] and premature monkeys [6]. Although %V_{10} varies considerably among animal species in comparison with %V_{20}, it does not appear to correlate with alveolar sizes (mean linear intercepts) [7]. %V_{10} has been determined to be influenced by lung injury in adult mouse models [3].
E, E_{resp}, and C, C_{resp}. These can also be indexed according to the pressure range used to determine the value.	Elastance and compliance of the respiratory system. The elastance is derived from the change in airway pressure that results from a change in lung volume. Compliance in the inverse of elastance. These values are derived from the slope of a linear model of a relatively flat portion of the deflation limb of the V-P curve, as the lung approaches the RV. When these metrics are generated using V-P data obtained during discontinuous deflation of the lung, they are referred to as *static* or *quasi-static* elastance and compliance. These parameters reflect, in part, the intrinsic elasticity of the entire respiratory system if it is measured *in situ* with an open thorax or *ex vivo* with isolated lungs. Accordingly, they are altered in lungs with parenchymal disease [3]. Historically, compliance is reported although elastance more accurately reflects the relationship between the independent (volume) and dependent (pressure) variables obtained during most lung mechanics studies.
E_{aw}<br>C_{aw}	This represents the elastance and compliance of the conducting airways. If performed using degassed lungs, it is thought that the first inflation arm of the V-P curve contains a portion where pressures generated by the lung distension reflect the distention of the conducting airways before the collapsed distal airways are recruited (P_{op}, [8]).
Hysteresis area	Area of the V-P curve bordered by the inflation and deflation limbs [2,9].

References:

1 Soutiere, S. E. & Mitzner, W. On defining total lung capacity in the mouse. *Journal of applied physiology* **96**, 1658-1664, doi:10.1152/japplphysiol.01098.2003 (2004).

2 Tankersley, C. G., Rabold, R. & Mitzner, W. Differential lung mechanics are genetically determined in inbred murine strains. *Journal of applied physiology: respiratory, environmental and exercise physiology* **86**, 1764-1769 (1999).

3 Limjunyawong, N., Fallica, J., Horton, M. R. & Mitzner, W. Measurement of the pressure-volume curve in mouse lungs. *Journal of visualized experiments : JoVE*, 52376, doi:10.3791/52376 (2015).

4 Johnson, J. W., Permutt, S., Sipple, J. H. & Salem, E. S. Effect of Intra-Alveolar Fluid on Pulmonary Surface Tension Properties. *Journal of applied physiology: respiratory, environmental and exercise physiology* **19**, 769-777, doi:10.1152/jappl.1964.19.4.769 (1964).

5 Morstatter, C. E., Glaser, R. M., Jurrus, E. R. & Weiss, H. S. Compliance and stability of excised mouse lungs. *Comparative biochemistry and physiology. A, Comparative physiology* **54**, 197-202, doi:10.1016/s0300-9629(76)80096-5 (1976).

6 Palmer, S., Morgan, T. E., Prueitt, J. L., Murphy, J. H. & Hodson, W. A. Lung development in the fetal primate, Macaca nemestrina. II. Pressure-volume and phospholipid changes. *Pediatric research* **11**, 1057-1063, doi:10.1203/00006450-197710000-00006 (1977).

7 Lum, H. & Mitzner, W. A species comparison of alveolar size and surface forces. *Journal of applied physiology: respiratory, environmental and exercise physiology* **62**, 1865-1871 (1987).

8 Robichaud, A. *et al.* Airway compliance measurements in mouse models of respiratory diseases. *American Journal of Physiology. Lung cellular and molecular physiology* **321**, L204-L212, doi:10.1152/ajplung.00470.2020 (2021).

9 Bachofen, H. & Hildebrandt, J. Area analysis of pressure-volume hysteresis in mammalian lungs. *Journal of applied physiology: respiratory, environmental and exercise physiology* **30**, 493-497, doi:10.1152/jappl.1971.30.4.493 (1971).

Table S2: LungElast software suite.

**Script that drives the device to collect the V-P data**	
LungElast.mpy	• Used to calibrate the pressure sensor or permit the use of default pressure calibration parameters or ones obtained during previous use of the script.<br>• Permits the user to test the leak of the system before the lung inflation studies.<br>• Moves the syringe piston to the home position, and then acquires V-P data using the maximum and minimum pressures, number of V-P cycles, motor movement parameters, etc., defined by Customizer.py and stored in customizations.json.<br>• Permits the user to determine the leak of the lung after the V-P data have been acquired.<br>• Saves the data to a file that can be downloaded by the user using a Wi-Fi or serial connection.<br>• Allows the user to continue with additional experiments or stop.
**Scripts that aid in setting up and testing the device**	
BusTester.mpy	• Used to test power to pressure sensor breakout board<br>• Used to test the i2c bus
DistVolCal.mpy	• Used to calibrate the locations of the lead screw nut ToF target at the minimum, home, and maximum positions.<br>• Used to calibrate the volume delivered by the syringe during advancement of the syringe plunger.
CompCal.mpy	• Used to obtain data that is employed by LungElast to compensate for pressure-dependent compression of the gas volume during a V-P experiment. After the LungElast system is built and the stopcock that would connect to the animal airway is closed, this script prompts the user to perform an experiment that allows the determination of how the change in system pressure changes the delivered volume. After the user determines the pressure-dependent volume compression, they enter the value into the system defaults using the Customizer script. The LungElast script then uses this factor to compensate for the pressure-dependent volume compression during a V-P experiment. This volume compression factor only needs to be redetermined when the syringe and conducting tubing of the system is changed.
Customizer.mpy	• Initially, prompts the user to adopt or customize LungElast control and V-P experiment options that are in defaults.py. The customizations are then stored in the customizations.json file.<br>• Subsequently, allows the user to edit the previous customizations saved in the customizations.json file or revert them to the ones in the default.py file.<br>• Gives the user the option to export the customizations in the json file to a file that is stamped with the date and experiment number and can be downloaded from LungElast.
**Required files used to set up the microprocessor**	
boot.py	• Turns off operating function debugging.<br>• Starts WebREPL, but note that WebREPL needs to be setup with its own password using the WebREPL_setup.py script that is encoded

	within MicroPython.
main.py	• Sets up device as Wi-Fi access point (AP) with ID ‘LungElast’<br>• Uses secrets.py to set AP password<br>• Instantiates HUZZAH32–ESP32 Feather Board red LED, pressure sensor board, and time of flight (ToF) sensor control GPIO pins<br>• Sets pins to low value so that they don’t float.
**Required files containing data that are used by other scripts**	
defaults.mpy	• This file contains default parameters that drive LungElast and their definitions<br>• It also has flags that control:<br>compression compensation<br>output of instructions<br>provision of parameter definitions.
config.py	• Contains GPIO and analog pin mappings for HUZZAH32–ESP32 Feather Board. They can be changed if another ESP32 breakout board is used.
secrets.py	• If the Wi-Fi is used, this file is used to contain the AP password that is employed by main.py to set up this feature.
**Required libraries that permit control of the ToF sensor and stepper motor and simple data analysis**	
Vl6180_LE_200.py	• Module originated by Radomir Dopieralski that was modified to permit the control and retrieval of ToF measurement data from the Vl6180 device via an i2c connection.
stepper.py	• Module by Tony DiCola / Adafruit that controls the stepper motor movement using the PCA9685 DC Motor and Stepper Driver Board controls via an i2c connection.
pca9685.py	• Module by Tony DiCola / Adafruit that is imported by stepper.py to and controls the PWM output from pca9685 chip using i2c and the attached stepper motor.
stats_LE.py	• Custom module that performs simple statistical analysis of data obtained from the pressure and ToF sensors.
**Files that will be generated by the above scripts**	
Customizations.json	• This file is generated by the Customizer script. It contains the user customizations that are used by the software to drive the device and obtain V-P data. The customizations are detailed in **Table** S3. This file is in json format and not meant to be directly modified or downloaded by the user.
V-P data files	• The LungElast script generates these files. The file name is formatted as follows: <experiment/subject number>_<date>.csv. The file contains the V-P data in columns with the following headers: subject, date, (V-P) cycle, stepCycles, stepNo, relstepVol, prescompVol, tofDist, and press. The user can download this data file via a hardwired or Wi-Fi link.
Customizer data files	• The Customizer script generates these files. The file name is formatted as follows: customizations_<date>_<experiment number>.txt The file contains the user-defined parameters that are defined using the Customizer script and that are used to drive LungElast and obtain the V-P data. The user can download the parameters via a hardwired or Wi-Fi link.

Table S3: LungElast control parameters. These variables can be defined by the user to control how LungElast obtains V-P data. They are initially supplied in the defaults.py file. But according to their particular device setup and experiment design, the experimentalist can accept or modify each them using the Customizer script. After the parameters are reviewed, they are stored in customizations.json for use by other scripts. By default, a definition of each parameter is provided to the user by the Customizer script at the time that they are reviewed.

Control	Parameter - Definition
Stepper motor movement	fstepNo. The number of steps made by the motor between pressure and ToF distance measurements driving the syringe plunger forward and injecting the gas into the lung.
	ascstepNo. The number of steps made by the motor near the peak pressure. Adjustment of this variable increases the resolution of the pressure determinations near the peak volume delivery into the lung and the determination of the volume that generates the desired maximum lung airway pressure.
	descstepNo. The number of steps made by the motor during the later phase of the deflation limb of the V-P determination. Adjustment of this variable increases the resolution of the pressure determinations near the final withdrawal of gas from the lung. This variable can be adjusted to enhance the modeling of the deflation V-P curve and the determination of the elastance of the lung.
	rstepNo. The number of reverse steps made by the motor between pressure and ToF distance measurements.
	hstepNo. The number of steps made to the home position between distance measurements.
V-P curve sampling	peakPresSamp. This is the percentage of the upper portion of the inflation curve during which smaller numbers of stepper motor steps are made so higher resolution V-P measurements are made. For example, a value of 10 means that higher resolution V-P measurements will be made within 10% of the maximum pressure defined by the user.
	endDeflatSamp. This is the percentage of the lower portion of the deflation curve during which smaller numbers of stepper motor steps are made so higher resolution V-P measurements are made. For example, a value of 30 means that higher resolution V-P measurements will be made during the lower 30% of the deflation curve as defined by (maximum pressure - minimum pressure).
Motor timing	presequalPause. This is the amount of time (in seconds) after the motor makes a train of steps and before the pressure reading is made. It allows the pressures to stabilize in the system.
	stepPause. This is a pause (in milliseconds) added after each step to compensate for the inertia of the lead screw nut.

Lead nut target distances	homeLoc. This is the distance (mm) of the lead nut screw target from the ToF sensor. It is related to distance at which the tip of the syringe plunger aligns with the maximum volume mark on its barrel.
	minLoc. This is the distance (mm) of the lead nut screw target at the point where the nut is closest to the ToF sensor.
	maxLoc. This is the distance (mm) of the lead nut screw target when the syringe plunger has been advanced to the point where it nearly fills the syringe barrel.
Pressure range	maxPres. This is the maximum pressure (cmH_2O) target.
	minPres. This is the minimum pressure (cmH_2O) target.
Pressure calibration	zero. This is the pressure sensor digital output during the atmospheric pressure calibration.
	slope. This value is determined during the pressure sensor calibration. The slope is used to convert the raw digital pressure sensor output to a calibrated pressure in cmH_2O.
Volume calibration	volCal. This is the volume of gas that is associated with 1 mm of syringe plunger movement. It is determined during syringe volume calibration. A NEMA 11 stepper motor with Tr5x2 produces 0.01 mm/step. Thus, 100 steps by the motor causes 1 mm of syringe plunger movement and delivers this volume of gas in µl. During the volume calibration of the syringe, water can be used instead of gas and the volume of water delivered by stepper motor-induced syringe plunger movement can be determined gravimetrically.
Compression calibration	slopeCor. This is proportional to the volume (µl) of gas compressed per cmH_2O gas pressure in the conducting system of the circuit. This parameter can be determined experimentally using CompCal.mpy.
V-P cycles	distpresCycles. This is the number of V-P loop cycles that are desired by the investigator.
Special system testing mode	maxTestPres. This is the maximum pressure target that will be achieved by LungElast during the testing of the device.
	testCycles. This is the number of V-P cycles that will be executed during the testing of LungElast.

Table S4. Parameters used during LungElast validation experiments with the elastomeric and mouse lungs. These parameters were set and reported using the *Customizer* script.

Soft elastomeric lung experiment parameter customizations – these were employed by LungElast while it obtained the data shown in **Figs.** 6a and S3:

```
motor timing
    stepPause      ....  20
    presequalPause ....   2
pressure range
    minPres        ....   0
    maxPres        ....  35
curve sampling
    endDeflatSamp  ....  30
    peakPresSamp   ....  30
vol calibration
    volCal         ....  40
compression correction
    slopeCor       ....   0
    offsetCor      ....   0
testing mode
    testCycles     ....   1
    maxTestPres    ....  35
motor movement
    fstepNo        .... 100
    descstepNo     ....  50
    hstepNo        ....  50
    ascstepNo      ....  25
    rstepNo        .... 100
cycles
    distpreCycles  ....   1
lead nut target distances
    maxLoc         ....  42
    homeLoc        ....  20
    minLoc         ....  16
```

Stiff elastomeric lung experiment parameter customizations – these were employed by LungElast while it obtained the data shown in **Figs.** 6a and S3:

```
motor timing
    stepPause      ....  20
    presequalPause ....   2
pressure range
    minPres        ....   0
    maxPres        ....  35
curve sampling
    endDeflatSamp  ....  30
    peakPresSamp   ....  30
vol calibration
    volCal         ....  40
compression correction
```

```
    slopeCor       ....   0
testing mode
    testCycles     ....   1
    maxTestPres    ....  35
motor movement
    fstepNo        ....  50
    descstepNo     ....  25
    hstepNo        ....  50
    ascstepNo      ....  25
    rstepNo        ....  50
cycles
    distpreCycles  ....   1
lead nut target distances
    maxLoc         ....  42
    homeLoc        ....  20
    minLoc         ....  16
```

P21 mouse pup lung experiment parameter customizations – these were employed by LungElast while it obtained the data shown in **Figs**. 6b and c and S4:

```
motor timing
    stepPause      ....  20
    presequalPause ....   2
pressure range
    minPres        ....   0
    maxPres        ....  35
curve sampling
    endDeflatSamp  ....  0
    peakPresSamp   ....  0
vol calibration
    volCal         ....  40
compression correction
    slopeCor       ....   0
testing mode
    testCycles     ....   1
    maxTestPres    ....  35
motor movement
    fstepNo        ....  100
    descstepNo     ....  100
    hstepNo        ....  100
    ascstepNo      ....  100
    rstepNo        ....  100
cycles
    distpreCycles  ....   1
lead nut target distances
    maxLoc         ....  43
    homeLoc        ....  20
    minLoc         ....  15
```

Bill of Materials

Designator	Component	#	Cost / unit USD	Cost each USD	Source of materials
Micro-processor breakout board	HUZZAH32–ESP32 Feather Board with ESP32 WROOM32 4 MB SPI flash 802.11BGN HT40 WiFi transceiver with antenna	1	20.95	20.95	Adafruit (pre-soldered plain headers version) 3591
Stepper motor control breakout board	DC Motor + Stepper FeatherWing	1	21.50	21.50	Adafruit 3243
ToF sensor breakout board	VL6180X Time of Flight Sensor	1	13.95	13.95	Adafruit 3316
Breakout interconnect-or board	FeatherWing Tripler Mini Kit	1	8.50	8.50	Adafruit 3417
Sensor controller board	Prototyping board	1	4.95	4.95	Adafruit 2884
LED, D1	5 mm LED	1	8.00 / 25	0.32	Adafruit 300
Resistors, R1-R4	1/8 W, +/- 5% value	4	15.99 / 2425	0.03	Amazon ASIN B01MDJQ1II
Transistor, Q1	PN2222A NPN bipolar transistor	1	0.20	0.20	Adafruit 756
Pressure sensor	All Sensors DLC-L20G-U2, 0 – 51 cmH_2O gauge male	1	35.73	35.73	Digi-Key 442-1202-ND
Perfboard for pressure sensor adapter	Copper strip board	1	8/10	0.80	Amazon ASIN B088GSJM7G
Perfboard for pressure sensor	Perfboard	1	4.95/10	0.49	Adafruit 2670

breakout board					
Headers, J1-J5	Break-away Male 0.1” headers	1	4.95/10 36-pin strips	0.49	Adafruit 3009
Jumper wires	Female / Female jumper wires	8	1.95/20	0.78	Adafruit 1950
USB cable	USB A-plug to Micro-USB	1	4.95	4.95	Adafruit 2185
Wire	22 AWG flexible 2 conductor wire	3’	0.60/ft	1.80	Tuofeng 12AWG<br>Amazon ASIN B0981NF1HV
Stepper motor, M	NEMA 11 non-captive, bipolar linear stepper motor	1	32	32	Stepperonline 11N13S0754AA-100RS
Battery switch, S	Tactile On/Off switch with leads	1	3.95	3.95	Adafruit 1092
Batter connector	JST PH 2-pin Cable, female connector	1	0.75	0.75	Adafruit 261
Battery, BAT	3.7V Lithium-ion polymer battery, 2200mAh	1	9.95	9.95	Adafruit 1781
**LungElast gas conductors**					
Glass syringe	1mL glass syringe, Metal Luer Lock	1	14.06	14.06	Air-Tite 7.140-21; Grainger 19G357
Lubricant	Super Lube-21030	1	6.67	6.67	Amazon ASIN B000XBH9HI
Tee Luer lock connector	Luer Tee Female x 3	1	28.75/25	1.15	Masterflex 45502-56; VWR MFLX45502-56
Straight Luer lock connector	Straight, Luer lock male x male with swivel	1	9.90	9.90	Cole-Parmer SK-06464-90

Luer lock to barb connector	Male Luer to hose barb 3/32" ID	1	22.25/25	0.89	Masterflex 30800-28; VWR MFLX30800-28
Tubing	Flexible PVC, 3/32" ID x 5/32" OD	0.3’	39.00/50ft	0.23	Masterflex 95505-02; VWR MFLX95505-02
Stop-cock	Four-way stopcock, male Luer	1	42.75/10	4.28	Masterflex 30600-12; VWR MFLX30600-12
**3D printed objects**					
PLA filament	PLA 1.75mm dia filament		32.99 / 1.00kg	0.33	Grainger 40LG47
Screws	M2.5 6 - 10 mm	6		0.30	Many sources
Screws	M2 x 10mm	2		0.10	Many sources
Knurled nuts	M2 x 4mm x 3.5mm	2		0.10	Many sources

Total cost each device: $200.10

Elastomeric model lung construction

Jesse Roberts Jr.

Aug 2, 2022

Introduction:

- An elastomeric model lung is useful for testing LungElast and for educational purposes.
- Below I describe how to construct two elastomeric model lungs, one that is stiff and relatively inelastic and another that is soft and relatively elastic.
- The elastomeric model lung consists of two parts: the elastomeric lung part made of latex and an adapter portion.
- The adapter portion is formed from two parts that are made using fused deposition modeling (FDM) 3D printing and polyactic acid (PLA; see Fig. on right).
    - The Luer end is the part of the adapter that connects to a male Luer connector on the LungElast device.
    - The bulb end is the part over which the elastomeric lung is stretched.
    - After these two parts of the adapter are printed, they are fused together, impregnated with epoxy, adhered to the elastomeric lung, and then attached to the LungElast device.

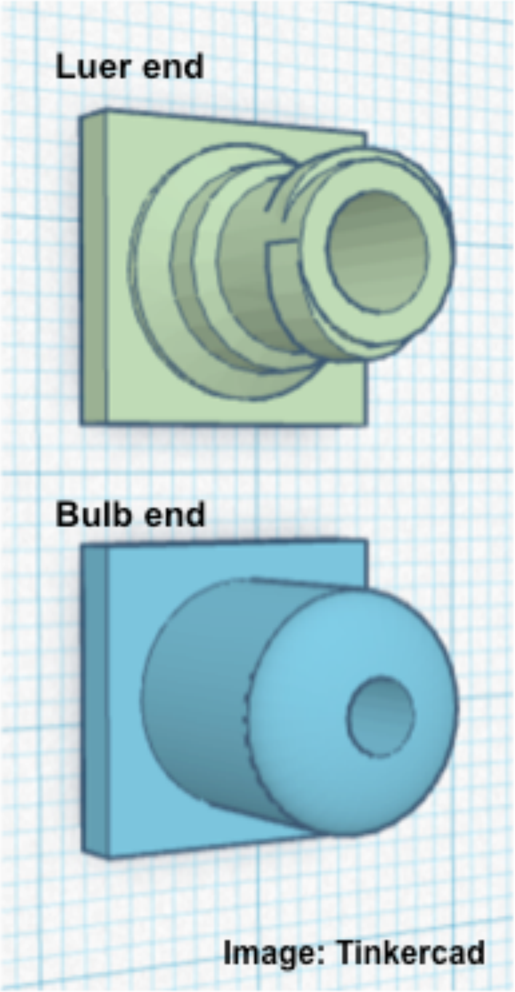

Materials:

- Elastomeric lung parts:
    - For stiff elastomeric lung –
        - 1.4 mm thick rubber pipet bulb
        - e.g. FSHR Bulb Red 2 ml, Cat. No. 03-448-27
    - For soft elastomeric lung -
        - 0.07 mm thick latex finger cot –
        - Small size finger cot
        - e.g. Amazon Standard Identification Number (ASIN): B001F5YRD0
- Adapter parts:
    - For stiff elastomeric model lung –
        - stiff_lung_bulb_end.stl
        - stiff_lung_Luer_end.stl
    - For soft elastomeric model lung
        - soft_lung_bulb_end.stl
        - soft_lung_Luer_end.stl
- Acetone
- Penetrating epoxy
    - e.g. TotalBoat TB-CPEKOTP
- Adhesive
    - e.g. 3M General Purpose 45 Spray Adhesive
- 4-way stopcock with Luer connections; male lock
    - e.g. Cole-Parmer 3060004, or ASIN: B003NV1MAU
- 10 ml Luer-lock syringe
- Tire bead sealer
    - e.g. Xtra-Seal 14-101, ASIN: B000GKD722
- Wooden applicator
    - e.g. Bamboo wooden toothpicks, ASIN: B0931QGLRN

- Gloves

Method:

1. Print and fuse the adapter parts
    a. Use the stl files to make 3D prints of the Luer and Bulb ends using PLA.
    b. Clean the orifices using a think wooden applicator.
    c. Use a syringe with a male Luer end and key the thread of the Luer end of the print
        i. Be careful not to cross-thread the connector
    d. In a chemical hood, apply acetone to the mating surfaces of the adapters.[i]
    e. Gently press the mating surfaces of the 3D prints together, with a slight twist so that the acetone-softened surfaces adhere well together. The mated parts should be assembled as shown in the Fig. below.
    f. Sight down the long axis of the prints to confirm that the center bore of the joined adapter pieces is aligned.
    g. Allow the two pieces to fuse.[ii]
2. Seal the adapter
    a. In a chemical hood, mix the two part penetrating epoxy together in a small bowl according to the manufacture's instructions.
    b. Paint the surface of the Luer connector and allow the epoxy to be adsorbed into the material. Be sure to avoid getting epoxy on the Luer connector threads.
    c. After about 30 min, wipe off the epoxy that was not adsorbed.
    d. When the epoxy that is leftover in the mixing bowl hardens, then the Luer connector is ready for the next step. This might take overnight.
3. Apply the elastomeric lung over the bulb end of the adapter
    a. In a well-ventilated area, spray the bulb end of the adapter with adhesive.
    b. After the adhesive is tacky, roll the elastomeric lung over the bulb.
    c. Remove any adhesive that has squeezed out.
    d. Let the adhesive cure.
4. Use a sealer to join the female Luer of the connector to the male Luer of the 4-way stopcock
    a. Connect the male Luer part of a syringe to a female Luer port of a 4-way stopcock.
    b. Using the wooden applicator, smear the bead sealer on the inside of the female Luer of the elastomeric lung adapter.
    c. Insert the male Luer of the stopcock into the female Luer of the elastomeric lung connector and gently twist the male lock to help smear the sealer on the mating surfaces of the Luer connections.
    d. Exercise the syringe—gently moving some air into and out of the Luer connector—to make sure that the sealer has not occluded the bore of the connector and stopcock.
    e. The completed assembly should look like the one shown in the Fig. below.
    f. Note: do not remove the 4-way stopcock from the elastomeric lung assembly as this will break the seal and likely cause air leakage
5. Test the elastomeric lung
    a. Attach the elastomeric lung assembly – 4-way stopcock to LungElast
    b. Perform a the leak test as directed by the LungElast script

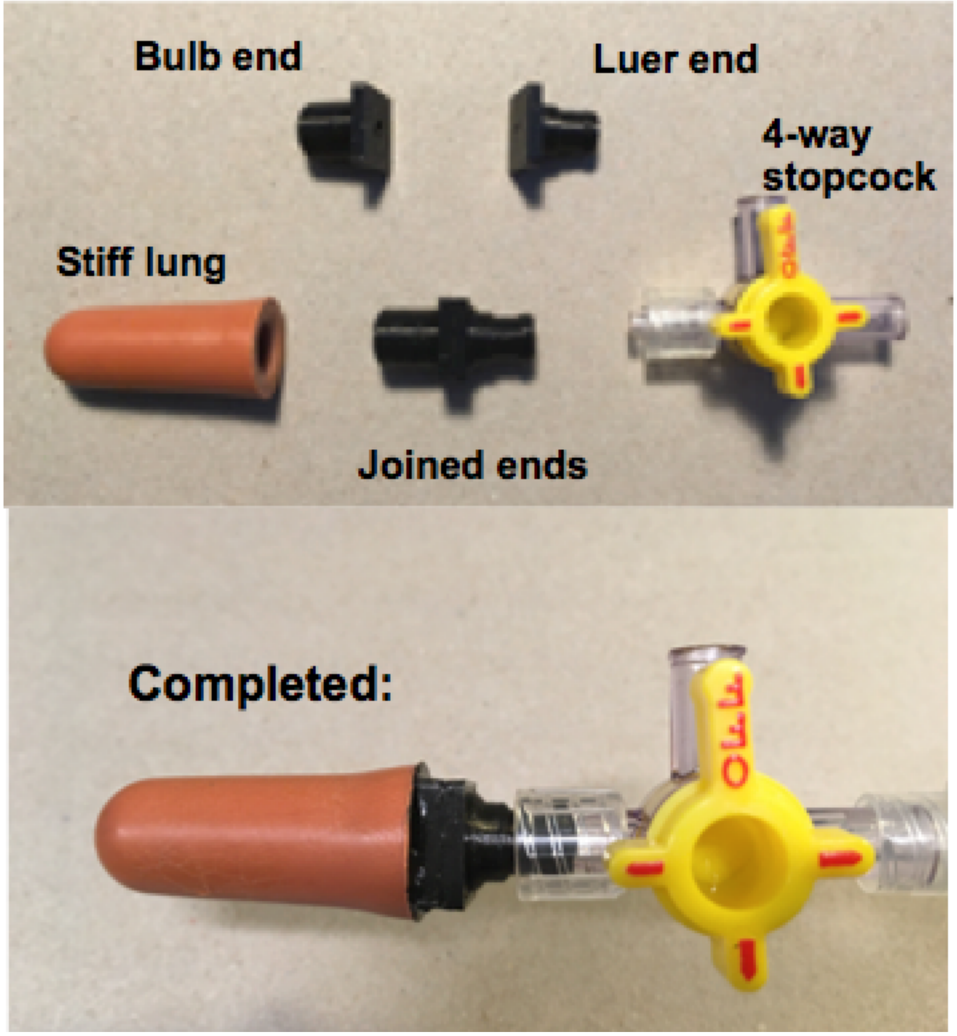

---

[i] I did not find that methyl acrylate formed a gas-tight connection between the two parts.
[ii] Note that the PLA might change color where the acetone came into contact with it. This does not affect the function of the connector.

ESP32 and MicroPython resources:

Information about the ESP32 microprocessor -
https://www.espressif.com/en/support/documents/technical-documents
https://www.espressif.com/sites/default/files/documentation/esp32_technical_reference_manual_en.pdf

Overview of the HUZZAH32–ESP32 Feather Board -
https://learn.adafruit.com/adafruit-huzzah32-esp32-feather

Schematic of HUZZAH32–ESP32 Feather Board -
https://learn.adafruit.com/adafruit-huzzah32-esp32-feather/downloads

How to establish a serial connection with the HUZZAH32 – ESP32 Feather Board -
https://learn.adafruit.com/adafruit-huzzah32-esp32-feather

Establish a serial communication with the ESP32 development board, general –

https://docs.espressif.com/projects/esp-idf/en/latest/esp32/get-started/establish-serial-connection.html

MacOS –Terminal serials communications program

https://www.imore.com/how-use-terminal-mac-when-you-have-no-idea-where-start

Windows and Linux – PuTTY SSH Client serial communications programs

https://www.putty.org/

Get driver for onboard HUZZAH32–ESP32 Feather Board USB to TTL converter to communicate with ESP32

https://www.silabs.com/developers/usb-to-uart-bridge-vcp-drivers

Information about MicroPython -
https://docs.micropython.org/en/latest/reference/index.html

Information about the MicroPython ESP32 port -
https://docs.micropython.org/en/latest/esp32/quickref.html

How to use esptool to flash the MicroPython with the ESP32 firmware -
https://docs.espressif.com/projects/esp-idf/en/latest/esp32/get-started/index.html#introduction
https://docs.micropython.org/en/latest/esp32/tutorial/intro.html#esp32-intro
https://randomnerdtutorials.com/flash-upload-micropython-firmware-esp32-esp8266/

Where to download MicroPython v1.19 binary for ESP32 -
https://micropython.org/download/esp32/

MicroPython shells -

ampy –
https://github.com/scientifichackers/ampy

rshell –
https://github.com/dhylands/rshell

MicroPython IDEs -

Thonny – (this also accesses the MicroPython REPL shell)
https://thonny.org/blog/2018/06/05/thonny_and_micropython.html

uPyCraft –
https://dfrobot.gitbooks.io/upycraft/content/

Pressure Sensor Breakout Board Construction
Jesse D. Roberts Jr.
Sept 24, 2022

Materials:

Designator	Component	#	Cost / unit USD	Cost each	Source of materials
Pressure sensor	Amphenol DLC-L20G-U2, 0 – 51 cmH_2O gauge male	1	35.73	35.73	Digi-Key 442-1202-ND
Perfboard for pressure sensor adapter	Copper strip board	1	8/10	0.80	Amazon ASIN B088GSJM7G
Perfboard for pressure sensor breakout board	Perfboard	1	4.95/10	0.49	Adafruit 2670
Headers, J1-J5	Break-away Male 0.1" headers	1	4.95/10 36-pin strips	0.49	Adafruit 3009

General considerations:

- The Amphenol pressure sensor is only available in a 6-SMD, gull wing package. To facilitate its integration into a pressure sensor breakout board, below are instructions on how to make a carrier board for the sensor and how to install it in a standard perfboard using through-hole techniques.
- The pressure sensor board incorporates a LED in the power pathway so that the LungElast user can confirm the pressure sensor's energization by the sensor control board.

Make pressure sensor carrier board –

1. Lay the pressure sensor on copper strip board with 0.1 inch (2.54 mm) hole spacing and trace out an area that encloses one row of holes above and one row of holes that are below its feet, as shown in Fig. 1, below.
2. Remove the sensor, and then mark the traces between the holes in the middle, as illustrated (Fig. 2).
3. Use a rotary tool with a cut off wheel and carefully cut through the copper traces as shown in Fig. 3. Be careful not to cut too much into the board substrate, as this will weaken the carrier board.
4. Using the rotary tool and carefully cut the outlines of the carrier board as shown in Fig. 4. Make the cut in several passes so that the board is not overheated it its remaining copper traces lift.
5. Remove the carrier board from the remainder of the board (Fig. 5). Use the sides of the cut off wheel, an emery board, or fine-grit sandpaper to smooth board's

edges. Clean the copper strips using isopropyl alcohol, and then lightly sand it until the copper is bright.
6. Make two 1x3 headers using the breakaway male headers. Install their long legs into a breadboard and then place the carrier board on top of it, orienting the board so that the cooper strips are facing upward.
7. Apply a small amount of liquid flux to the cooper strips, the headers that are protruding through them, and the feet of the pressure sensor.
8. Apply solder to the copper strips and headers, as shown in Fig. 6, and then let it cool. Use a multimeter to confirm that there is electrical continuity between the solder-covered copper strips and the headers. Also, using the meter, make sure that there is no bridging of solder between the strips. If there is bridging, use a solder wick or sucker to remove some of the solder. However, make sure that some solder remains on the copper strips.
9. Carefully align the sensor so that its feet lie directly over the copper strips. Briefly touch the feet of the sensor with the solder iron tip so that the solder reflows and joins the feet to the copper strips, as shown in Fig. 7. Be careful not to heat the sensor feet for too long; this will melt the sensor housing and destroy it.
10. After the solder has cooled, remove the sensor carrier board from the breadboard and use a continuity meter to confirm that the header pins are electrically connected to the sensor feet and that there is no bridging. If bridging is detected, return the sensor carrier board to the breadboard, use a wick to remove the excess solder, and reapply it if needed.
11. Clean the liquid flux from the board as directed by the manufacturer’s instructions.
12. The resulting pressure sensor carrier board is shown in Figs. 8 and 9.

Make pressure sensor breakout board –
1. Solder the sensor carrier board, LED, resistor, and header to a perfboard as directed by the schematics.
2. Check the connections for electrical continuity using a multimeter.
3. The top of the board should look like what is shown in Fig. 10. Note that I painted the positive pins of the pressure sensor carrier board and the breakout board red to aid in their construction and use.

Pressure sensor breakout board construction - Figs

Fig 1.

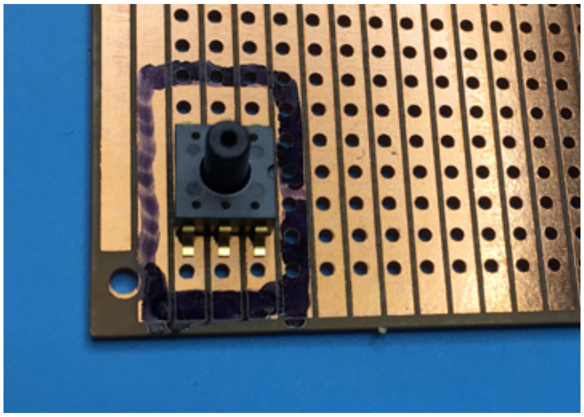

Fig 2.

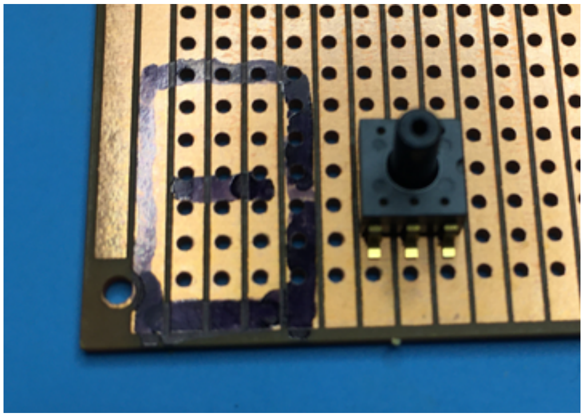

Fig 3.

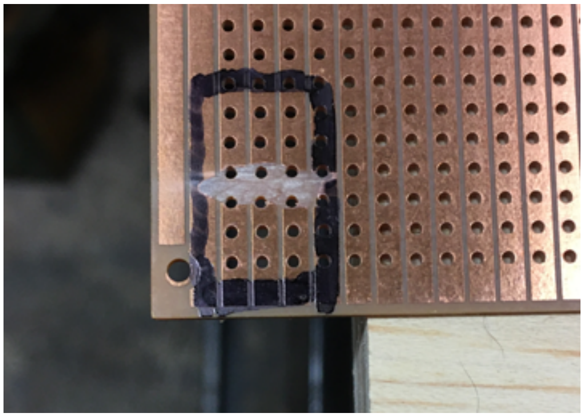

Fig 4.

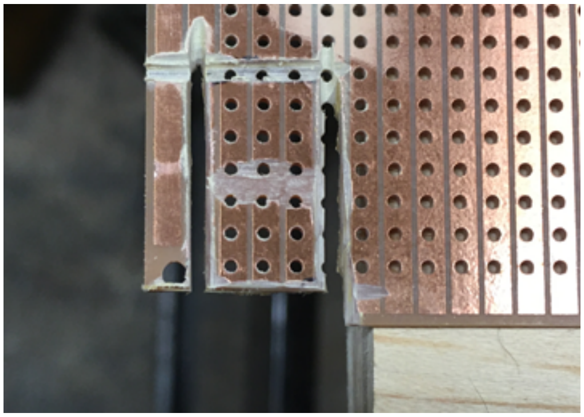

Pressure sensor breakout board construction - Figs

Fig 5.

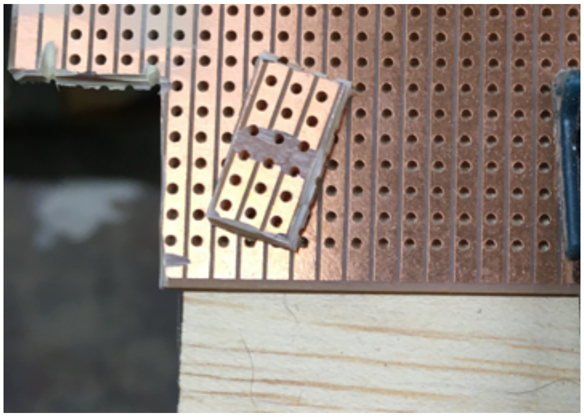

Fig 6.

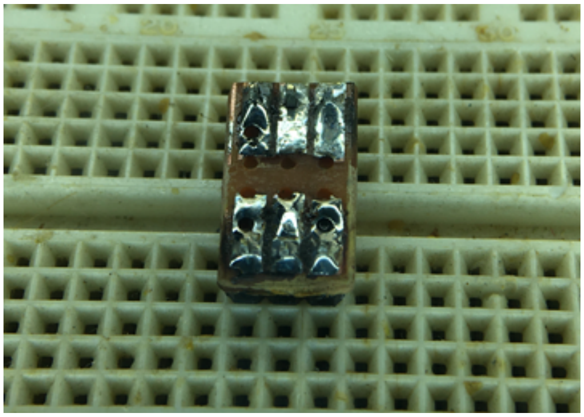

Fig 7.

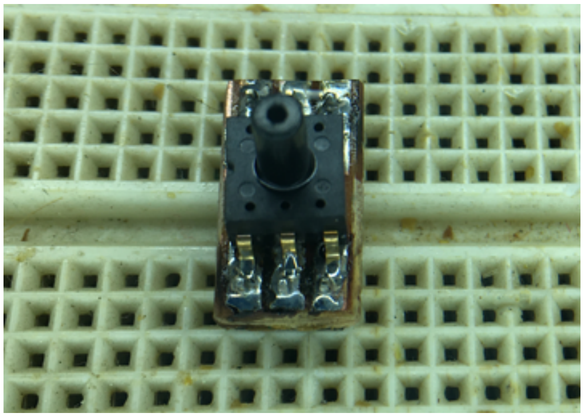

Fig 8.

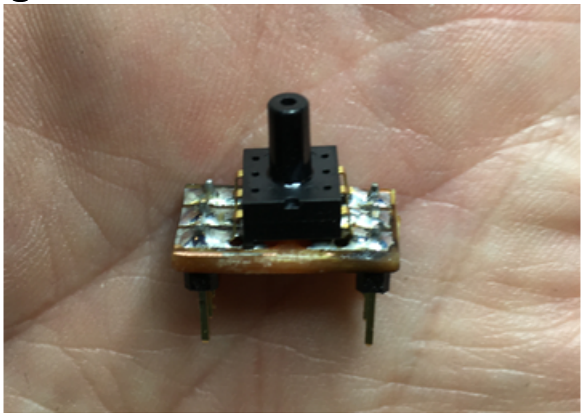

Fig 9.

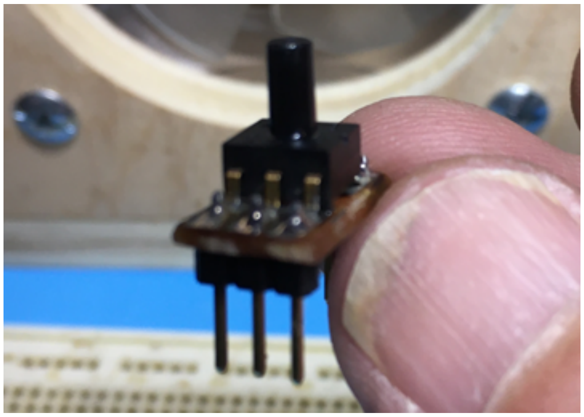

Pressure sensor breakout board construction - Figs

Fig 10.

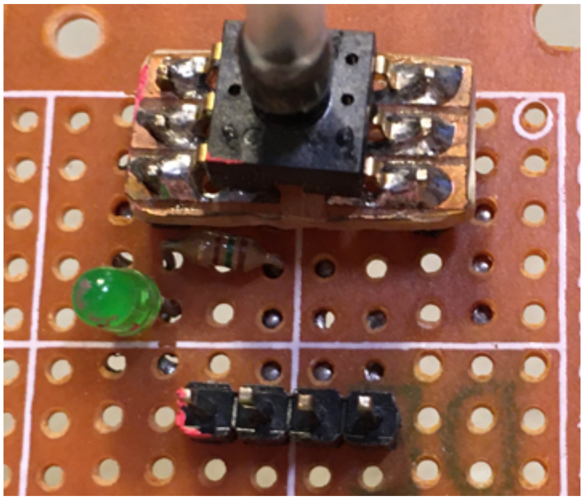

Pressure sensor breakout board wiring *Roberts*

Top side -

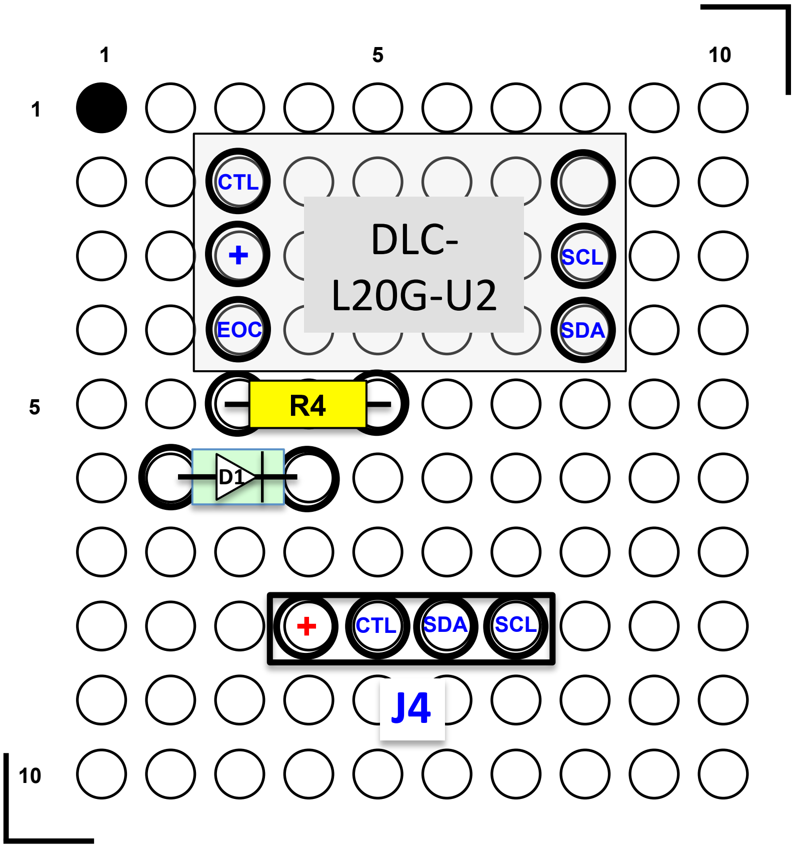

Notes:

- The surface-mount feet of the Amphenol DLC-L20G-U2 pressure sensor are soldered onto a custom adapter board with two sets of 1x3 connectors. This adapter board is then soldered directly onto the pressure sensor breakout board or joined to 1x3 female connectors that are soldered on the board. The later configuration allows flexibility in changing the sensor if desired.
- The J4 1x4 male connector is shown on top side of board, but it could be soldered to bottom side, if more convenient.

Pressure sensor breakout board wiring *Roberts*

Bottom side -

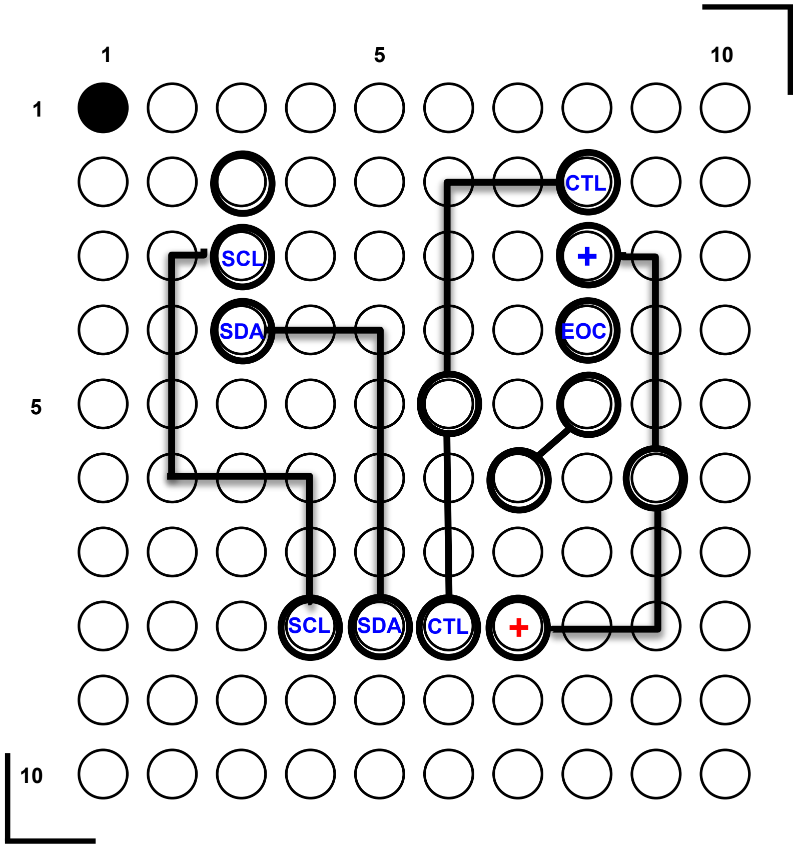

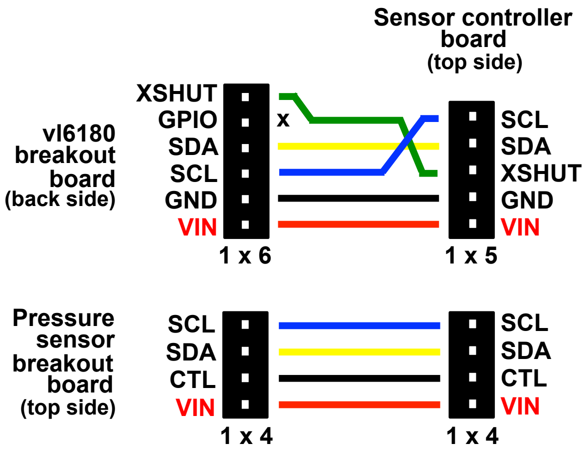

LungElast cables. Female 2.54mm pitch connectors with ~20cm of 28AWG wire. An easy way to make the custom ribbon cables is to buy premade ones that are of the desired length and terminate in 1x1 housings, remove the extra wires until 4 wire-width cables result, un-plug the 1x1 housings, and then re-plug the terminals into the 1x6. 1x5, and 1x4 housings as shown above.

Sensor controller board wiring

Roberts

Top side -

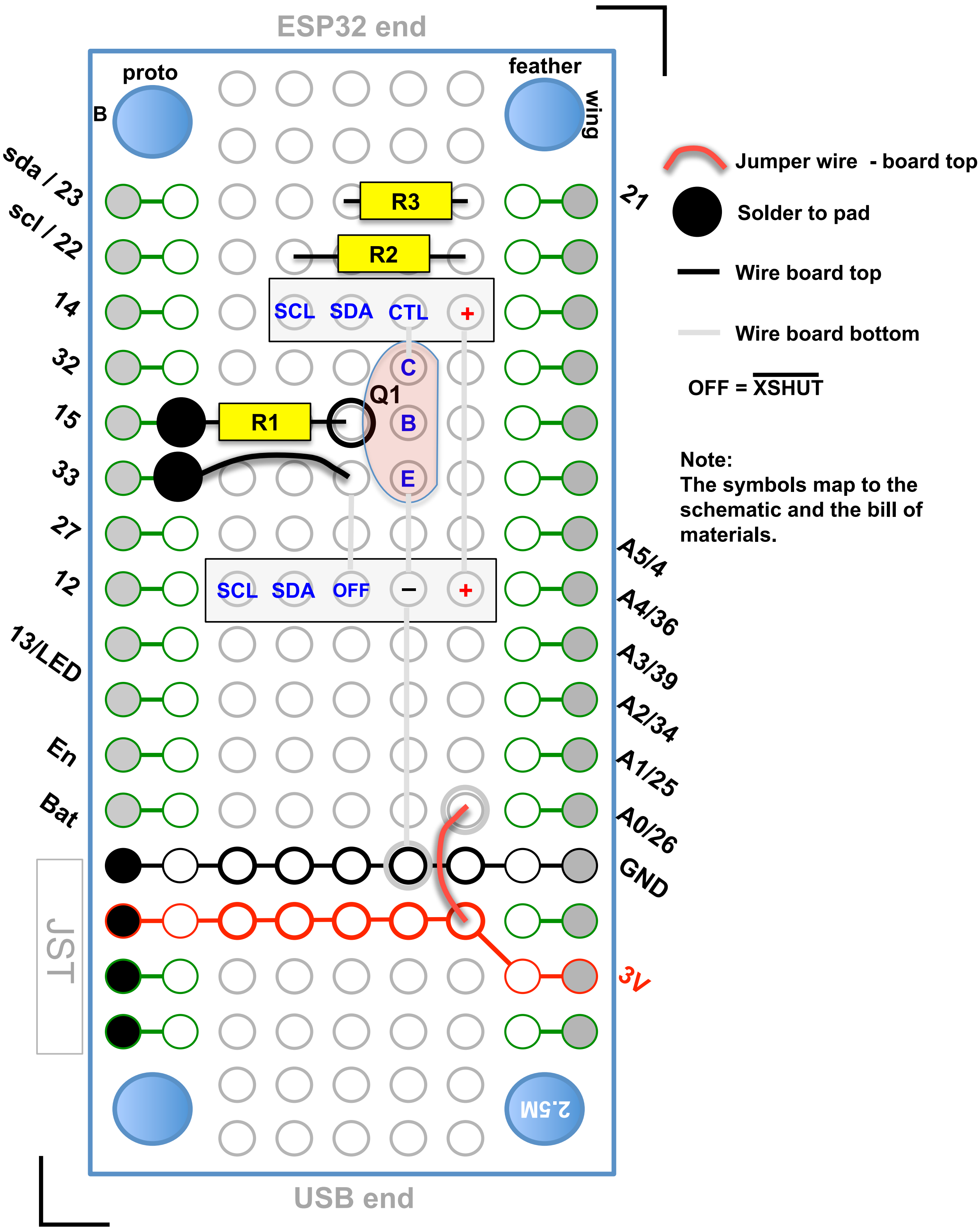

Note:
The symbols map to the schematic and the bill of materials.

Sensor controller board wiring

Roberts

Bottom side -

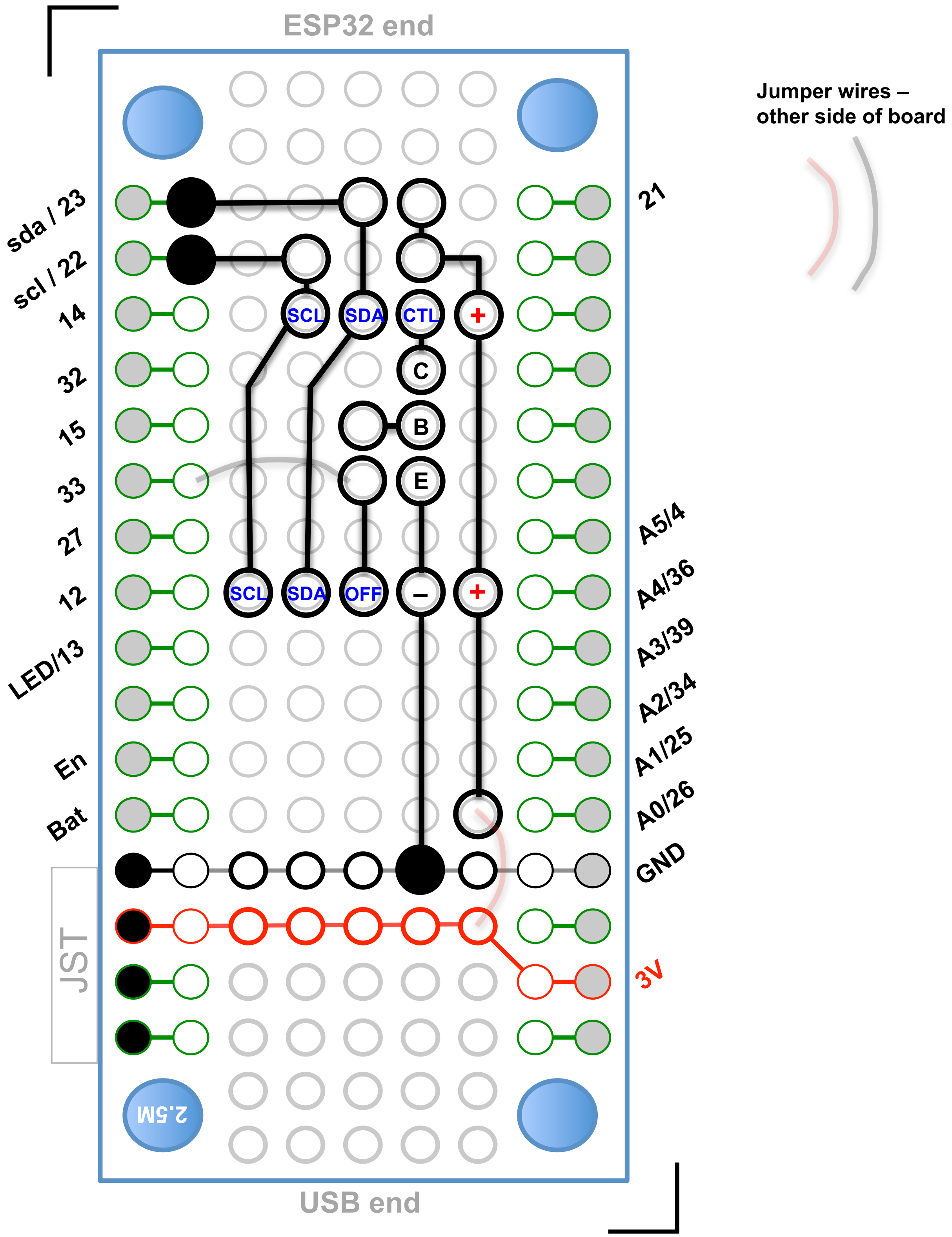

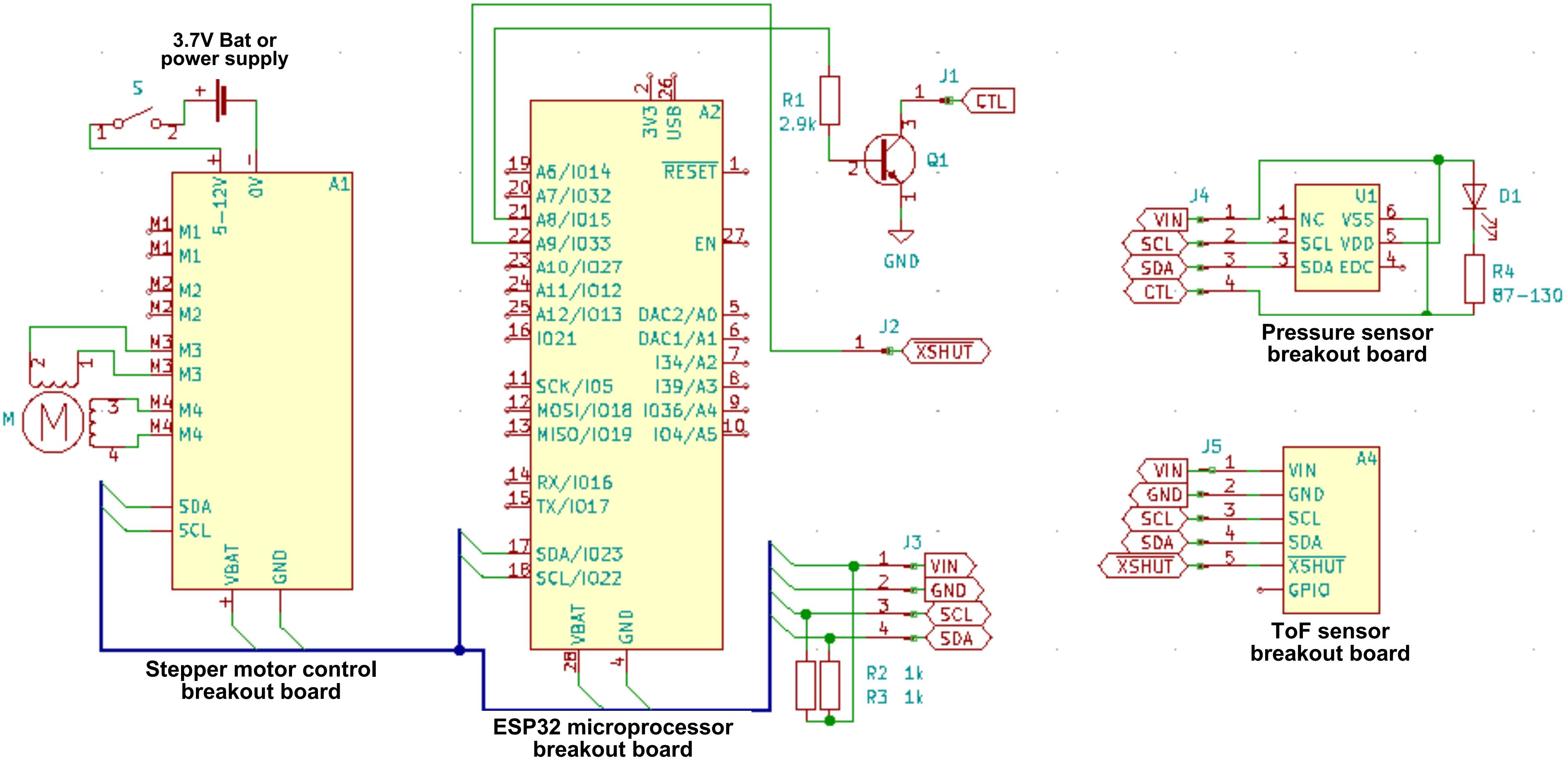

LungElast Schematic. The components are M, NEMA 11non-captive, bipolar linear stepper motor; S, On/Off switch; R, resistors; Q, PN2222A NPN transistor; D, LED diode. See the *Bill of Materials* for their sources.
